# Wide field of view multifunctional solar sensor for photovoltaic power management via measurement of solar angle and intensity

**DOI:** 10.1038/s41378-025-01154-4

**Published:** 2026-02-11

**Authors:** Yifeng Liu, Qingfeng Wu, Haizhao Feng, Yier Xia, Minghao Xu, Sixing Xu, Xiangyu Zhao, Philippe Basset, Xiaohong Wang

**Affiliations:** 1 School of Integrated Circuits, Tsinghua University, Beijing, China; 2 College of Semiconductors, Hunan University, Changsha, China; 3 Univ Gustave Eiffel, CNRS, ESYCOM, Marne-la-Vallée, France

**Keywords:** Engineering, Optics and photonics

## Abstract

Large-scale photovoltaic systems are a rapidly expanding contributor to sustainable energy production, and power management for these systems relies on measuring both solar angle and intensity simultaneously. However, current non-miniaturized sensors often offer a narrow field of view and measure only a single parameter, which does not meet the needs of advanced integrated photovoltaic power-management systems, motivating the need for a compact, multifunctional sensing solution. We propose a new, integrated, multifunctional sensor capable of capturing wide-view solar angle and intensity. This device integrates three detectors on a single chip, each with a differently inclined surface, to broaden the field of view. Tests under systematically varied angles and intensity levels showed that the three detectors respond most strongly at 117.5°, 87.5°, and 67.5°, with current-to-intensity coefficients of 2.85 × 10^−^^4^, 2.31 × 10^−^^3^, and 2.57 × 10^−^^4^ μA/(W/m^2^). The device offers an unprecedented ±75° field of view for a single-chip solar sensor while maintaining a low mean error of 3.4° for the angle and a low relative mean error of 1.6% for intensity, respectively. This multifunctional micro-electro-mechanical system (MEMS) sensor, combining a wide field of view with high accuracy, marks an important step toward enabling distributed, in-situ power management in large-scale photovoltaic systems.

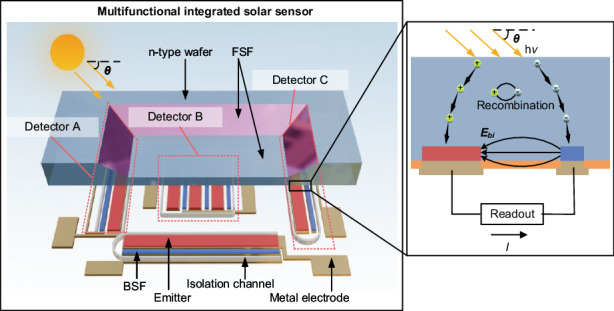

## Introduction

Large-scale photovoltaic (PV) systems contribute significantly to more sustainable sources of energy^1–3^. PV cell architecture and materials have steadily increased the solar conversion efficiency. However, these increases from changes to the PV cell are rapidly approaching physical and material limits^4–7^. Accordingly, maximizing energy yield of large-scale PV systems increasingly depends on power management methods, such as solar tracking, maximum power point tracking (MPPT), and photovoltaic array reconfiguration (PVAR).

Solar tracking aligns PV panels with the sun’s trajectory to maximize incident light reaching the panel, thereby increasing energy yield^8,9^. In power electronics, MPPT algorithms optimize the operating point of each PV panel to maximize energy transfer under varying intensity and temperature conditions^10^. At the system level, PVAR modulates connections among PV modules to mitigate mismatch and shading losses, which are inevitable in large-scale installations^11^. These power management techniques are complementary and are often integrated into a comprehensive photovoltaic power management system, where their effectiveness depends on accurate measurements of solar parameters, especially solar angle and intensity^12,13^.

The solar angle can be derived either by astronomical-based algorithms^14^ or by sensor-based systems^15^. While the astronomical-based algorithms perform well under ideal conditions, their dependence on exact initial inputs (e.g., location, elevation, time, etc) and lack of real-time sensing and control reduce their effectiveness when installation misalignments, model drift, or transient environmental effects are present^16^. Sensor-based tracking provides real-time feedback and control, compensating for misalignment and environmental effects while enhancing system reliability and performance^17^. Similarly, solar intensity measurement improves the effectiveness of MPPT and PVAR, but it is commonly estimated indirectly by monitoring the output of the PV array^18,19^. This indirect approach provides only approximate values that reflect the aggregate response of the series-connected PV array without being able to resolve more localized panel and environmental differences, thereby limiting the effectiveness of PV system optimization techniques^20^. Additionally, because solar angles and intensity vary locally due to shading, soiling, or structural factors, compact distributed in-situ sensors are central to effective power optimization and responsiveness to localized environmental changes in large-scale PV systems^21^.

Some sensors have been developed to meet this need. For instance, sun-pointing sensors that use light and shade on photodetectors to calculate the sun’s position^16^. However, their lower precision, sensitivity to weather, and complex control algorithms limit their practical use. Collimating sensors use a tube or other structures to narrow the sun’s rays for high precision in a compact design, but at the cost of a narrow field of view (FOV), complex control systems, need for specialized materials, and frequent calibration^22^. Tilted mount photo-sensors achieve a wide FOV and high sensitivity through a pyramidal structure of photodetectors, yet their high cost, complex hardware, and calibration requirements make them difficult to scale. Collectively, drawbacks of large form factor, high power consumption, limited scalability, and the separation of angle and intensity measurement represent challenges to the practical use of solar sensors^23^.

The development of highly integrated Micro-Electro-Mechanical System (MEMS) based solar sensors is essential to overcome existing challenges, yet only a few single-chip devices have been reported. Joost et al. designed a compact, 10 mm × 10 mm sensor chip on a SiC wafer, which operated under extreme conditions, but was hampered by computational complexity and a limited FOV of ±37°^24^. Chaowanan et al. developed an ultraviolet solar sensor that expanded the FOV to ±55° but exhibited a high angular deviation of 10°^25^. In contrast, Liu et al. achieved a very low deviation of 0.3° but at the cost of a reduced FOV of ±48°^26^. These examples highlight the tradeoff between FOV and accuracy when miniaturizing solar sensors. The FOV is an essential parameter because it determines the range at which a sensor can precisely detect the sun’s position. A sufficiently wide FOV is necessary to capture the sun’s trajectory throughout the day and accommodate misalignments or installation errors without requiring mechanical movement. Existing designs, however, face tradeoffs between FOV, accuracy, and manufacturability, and most sensors measure only angle, neglecting solar intensity. Consequently, there is a clear need for compact, wide-FOV sensors that integrate angle and intensity measurement on-chip to improve the efficiency and scalability of large-scale PV systems.

To address these limitations, we present a multifunctional integrated solar sensor (MISS) capable of simultaneously measuring solar angle and intensity. The device integrates detectors with differently inclined surfaces on a single chip, expanding the field of view. The sensor is realized with MEMS technology, enabling miniaturization and scalability. Systematic characterization of the device establishes the relationships between detector responses and the angle and intensity, and these relationships are used to determine angle and intensity from detector outputs with high accuracy.

## Device design and fabrication

### Device design

**Fig. 1:**
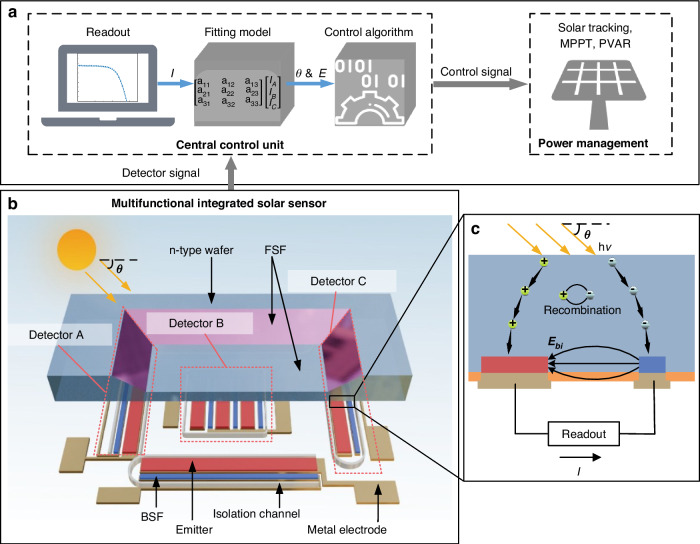
Overview of the MISS system integration, design, and operating principle. **a** Schematic diagram of a sensor-enabled power management system for large-scale photovoltaics. **b** 3D model of the silicon-based MISS device. **c** The operating principle of the detector

As mentioned in the introduction, a comprehensive power management system for large-scale photovoltaic arrays consists of sensing units, a central control unit, and a power management system (Fig. 1a)^12,13^. Sensing units are deployed throughout the PV system to capture localized solar angle and intensity data. Readout and communication circuits relay the collected data to the central control unit, where a fitting model and control algorithm process it to generate control signals. These signals regulate multiple aspects of the PV system, including motors for solar panel tracking and switches or relays for PVAR and MPPT. Within this architecture, the MISS serves as the core sensing element, providing compact, wide-FOV, in-situ measurements that enable real-time control of the PV system.

The MISS, fabricated on a silicon substrate, is composed of three detectors. Its 3D structural model is shown in Fig. 1b. An interdigitated back-contact (IBC) solar cell architecture is used for the detectors, which maximizes photoelectric conversion efficiency by arranging both the positive and negative electrodes in an interdigitated pattern on the back of the cell^27^. A key advantage of the IBC architecture is that it allows each detector’s front surface to be modified to have different geometries, a feature that is exploited to increase the FOV.

Leveraging this structure, the detectors operate on the photovoltaic effect, where incident light is converted into a measurable electrical signal (Fig. 1c). When light strikes the front surface, photons are absorbed by the silicon, generating electron-hole pairs^28^. The front surface field (FSF) on the surface of the device effectively reduces surface recombination of photogenerated carriers and facilitates their movement towards the back electrodes^29^. At the same time, the back surface field (BSF) and emitter enhance charge separation and collection, establishing a built-in electric field that drives carriers to the corresponding electrodes^30^. This controlled migration of charge carriers produces a current that is conducted by the metal electrodes.

The photocurrent of a detector is closely associated with the solar angle and intensity. The solar angle, $\theta$, is defined in accordance with the conventions illustrated in Fig. S3, with 0° along the left horizontal, 90° vertical, and 180° along the right horizontal. If the unit vector of the incident light and the normal vector of the illuminated surface are $\mathop{L}\limits^{ \rightharpoonup }$ and ${\vec{n}}_{i}$, the photocurrent (${I}_{i}$) of the detector can be expressed as follows^31,32^:

1 $${I}_{i}={\kappa }_{i}{E}_{i}{S}_{i}\cdot \left({\vec{n}}_{i}\cdot \mathop{L}\limits^{ \rightharpoonup }\right)$$

where ${\kappa }_{i}$ is a material-dependent coefficient. ${E}_{i}$ and ${S}_{i}$ are the intensity of the incident light and the illuminated area of the detector, respectively. Detectors with differently inclined active surfaces generate different responses under the same illumination. Combining the response currents of three detectors and using a physics-based fitting model, the solar angle and intensity can be accurately determined.

Simulations of the photovoltaic effect in the chip are provided in Sections S1–4 of the Supplementary Information. The differently inclined illuminated areas of the three detectors are formed using an anisotropic KOH etching process. A 400 μm wafer thickness and a KOH etch depth of 250 μm are selected to balance electrical performance and mechanical integrity during KOH etching. Simulations were also performed to determine the dimensions of the p-n junction. As shown in Fig. S4, device performance is largely independent of junction depth but exhibits a positive correlation with p-junction width and a negative correlation with n-junction width. Since the total width for detectors A and C is limited to 175 μm by the projected area of the KOH etching, the p-junction widths are set at 110 μm, and an n-junction width of 10 μm. Simulations of the implantation dose indicate the optimal ion implantation doses for the FSF, BSF, and emitter to be 2 × 10¹² cm⁻², 4 × 10¹⁵ cm⁻², and 3 × 10¹⁶ cm⁻², respectively (Fig. S5). However, a cost-reduced combination of 2×10¹² cm⁻², 1 × 10¹⁵ cm⁻², and 1 × 10¹⁵ cm⁻² is selected for fabrication, trading a less than 4% performance loss for significantly lower process cost.

Isolation channels are implemented to suppress lateral carrier drift between detectors. Simulation indicates that sufficiently deep isolation channels suppress drift (Fig. S6). To avoid structural failure near KOH-etched inclines, a 50 μm isolation channel was adopted as a compromise between isolation performance and mechanical reliability. This 50 μm isolation channel depth is able to attenuate 87% of the lateral current drift, an acceptable level. The specific design parameters are displayed in Table S2 and Fig. S7.

### Fabrication

**Fig. 2:**
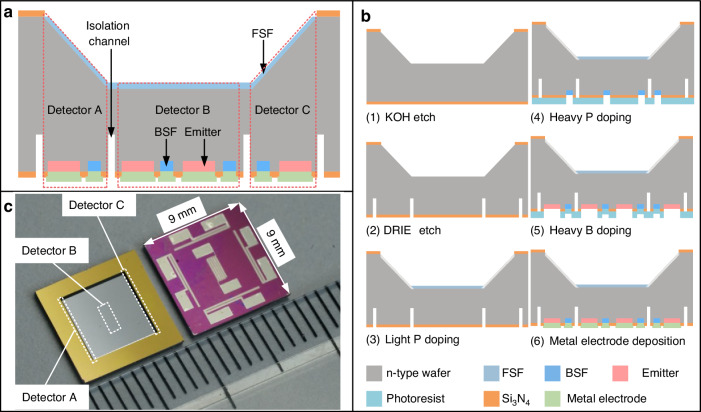
Fabrication process flow and optical photograph of the MISS. **a** The detailed cross-sectional view of the device. **b** The fabrication process of the device. **c** Top (left) and bottom (right) photographs of the MISS device fabricated on an n-type Si wafer

The MISS is lithographically microfabricated using a process involving five masks with processes on both sides of the wafer (Fig. 2a), with the fabrication process illustrated in Fig. 2b. The process starts with a <100> n-type silicon wafer, with 400 μm thickness and resistivity of 4 Ω cm (step 1). A thin Si_3_N_4_ layer with 100 nm thickness is deposited on both sides of the wafer with LPCVD to serve as a protective layer for KOH etching and an electrical isolation layer. Subsequently, patterning is performed on the front surface to define the KOH etching region. The subsequent DRIE etch process defines the p-n junction area and isolates the detectors (step 2). The FSF region is created by low-dose phosphorus ion implantation, with the implantation dose of 2 × 10^12 ^cm^−^^2^ and the implantation energy of 30 keV (step 3). The rear BSF and the emitter electrodes are formed by heavy doping with phosphorus (step 4) and boron (step 5), respectively. The implantation dose and energy for phosphorus ion implantation and boron ion implantation are both 1 × 10^15 ^cm^−^^2^ and 30 keV. To repair the implantation-induced lattice damage and activate dopants, the wafers undergo rapid thermal annealing (RTA) at 1000 °C for 10 seconds. After ion implantation, a 10/190 nm thick layer of Ti/Al is deposited on the wafer by evaporation and is patterned by liftoff (step 6). The fabricated wafer (Fig. S8) is then singulated into individual chips measuring 9 mm × 9 mm (Fig. 2c).

**Fig. 3:**
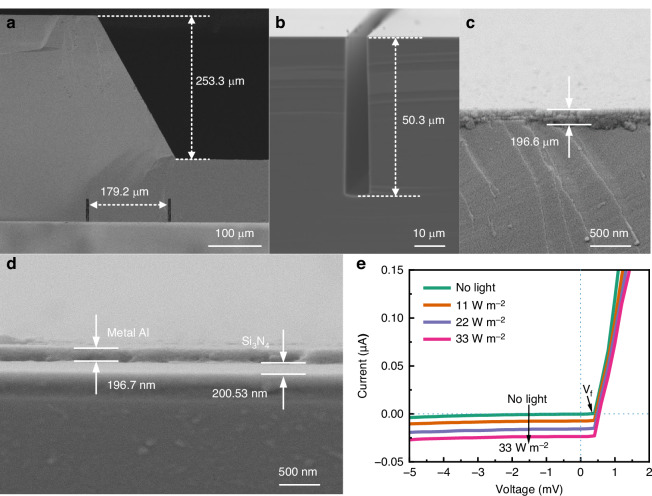
Structural characterization of critical positions for the MISS. The SEM images of **a** the corresponding position between the isolation channel and inclined surface, **b** the isolation channel, **c**, **d** metal over the p-n junction and silicon nitride isolation forming the metal connections and wire pads. **e** I-V curve of the fabricated photodiode under different illuminations

The detailed morphological characterization of the fabricated MISS is presented in Fig. 3. The cross-section profile of the detector surface and the rear isolation channel confirms that the p-n junction area aligns with the inclined surface of detector A. The measured KOH etch depth and the distance between two isolation channels on the inclined surface are 253.3 μm and 179.2 μm, respectively, matching the designed values. Furthermore, Fig. S9a and S9b reveal that the KOH etching angle is the expected 54.7°, showing that the three detectors in the MISS are inclined differently as designed. An enlarged view of the isolation channel region (Fig. 3b) shows the etch depth to be 50.3 μm, with bottom and top widths of 8.3 μm and 6.9 μm, respectively (Fig. S9c). Figure 3c and 3d illustrate the details of the deposited metal layers on the p-n junction and silicon nitride isolation areas. The metal thicknesses at two locations are 196.6 nm and 196.7 nm, respectively, indicating the successful formation of metal interconnects. Overall, the results of SEM images demonstrate that the fabricated structural details of the device are consistent with the designed parameters in Table S2.

The current-voltage (I-V) curves of the fabricated p-n junction under varying optical intensities are presented in Fig. 3e. The curve exhibits the expected photodiode behavior: a steeply rising current under forward bias and a minimal, saturated current under reverse bias. As the light intensity increases, the reverse current increases and the open-circuit voltage shifts to higher values, consistent with enhanced photocurrent generation. This behavior arises because incident light generates additional electron–hole pairs, which are separated by the built-in electric field, producing a photocurrent that adds to the diode current^33^. Given the extremely low concentration of minority carrier, the reverse current remains very small^34^. It can be observed that the short-circuit currents and open-circuit voltage of detectors A and B exhibit a clear increasing trend with intensity, which is a typical photodiode characteristic (Fig. S10).

## Experimental results and discussion

### Experimental results

The experimental test setup for characterizing the performance of the MISS is shown in Fig. S11. The test setup consisted of a simulated light source composed of LEDs that emit light across the solar spectrum with difusers to fully mix the light. The intensity was controlled by adjusting the power supply of the simulated light source, with the relationship between lamp power and intensity presented in Fig. S12. In addition, the device was mounted on a rotary stage to adjust the angle, and measurements of the angle were made using a digital inclinometer with an accuracy of 0.2°. The simulated light source was placed 10 cm away from the mounted chip, and all tests were conducted in a dark room. The intensity of light was measured using a lux meter with an accuracy of 1 W m^−^^2^. For each angle and intensity, the output currents from the three detectors were measured using a multimeter (Agilent 34411 A, Keysight Technologies, USA). The device was tested under various incident angles (ranging from 0° to 180° over 30 steps) and intensities (ranging from 100 W/m^2^ to 300 W/m^2^) to explore the relationship between the detector outputs and both angle and intensity.

**Fig. 4:**
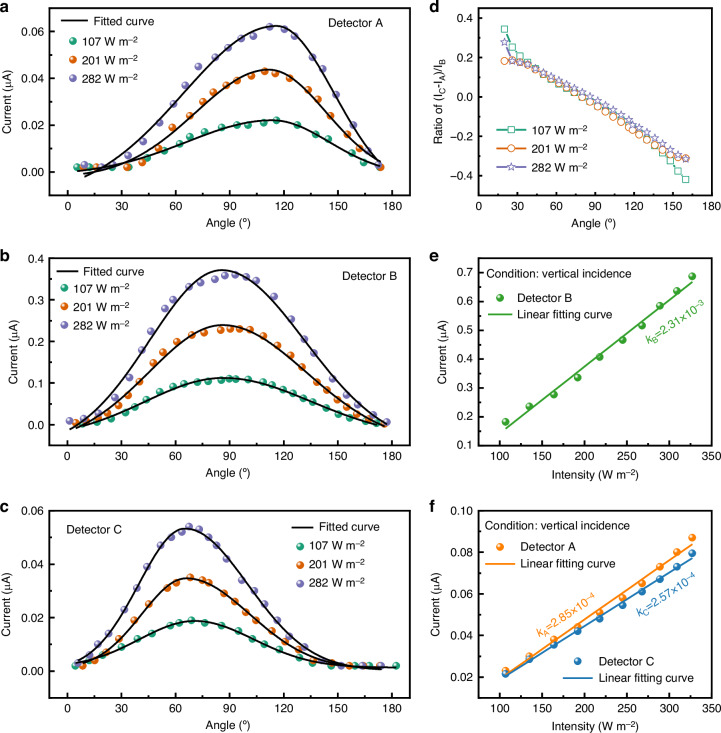
The test and analysis of the photoelectric current response for the MISS. **a**–**c** The relationship between the angle of incident light and the output current. **d** The relationship between D value and angle of incident light. **e**, **f** The relationship between intensity and the output current for three dectors with the light under vertical incidece

The three detectors exhibited similar responses under illumination at different angles. In all cases, the maximum output occurred when the incident light was perpendicular to the active surface, and dropped to low values at extreme angles, defining the sensor’s FOV (Fig. 4a–c). This trend was also maintained across different light intensities. The peak output of detectors A, B, and C occurs at 117.52°, 87.55°, and 67.54°, respectively. However, the theoretical maximum output of detectors A, B, and C should occur at 144.75°, 90°, and 35.25°, respectively, due to the angle formed by anistropic KOH etching. This discrepancy can be attributed to the significant number of carriers generated in the bottom area, which subsequently drift towards detectors on both sides and causing the shift in where the response maximum occurs^35^. To remove the influence of intensity *E* when calculating the solar angle, the parameter $D=\frac{{I}_{C}-{I}_{A}}{{I}_{B}}$ was defined. The value of *D* exhibited a monotonic relationship and overlaps across different intensities, showing that the angle can be determined independent of intensity (Fig. 4d).

Further investigation was conducted to explore the correlation between detector output and intensity. As shown in Fig. 4e and 4f, a linear relationship between intensity and output current was observed for the three detectors with the light under vertical incidence. This relationship is described by the variable *k* where *k*_A_, *k*_B_, and *k*_C_ represent the ratios of the generated photocurrent to intensity for detectors A, B, and C, respectively. Among them, *k*_A_, *k*_B_, and *k*_C_ are 2.85 × 10^-4^, 2.31 × 10^-3^, and 2.57 × 10^-4^, respectively. Due to the symmetrical structure of detectors A and C, their *k* values are very similar. Similar relationships were seen at angles of 60° and 120° (Fig. S13**)**. The persistence of the linear relationship at different angles shows that the intensity can be determined from the output of the detectors.

### Angle fitting analysis

To obtain the solar angle from the detector responses, a fitting model based on the geometry of the MISS was developed. The effective intensity of the incident light is calculated by multiplying the dot product between the detector surface’s unit normal vector and the incident illumination vector. The geometric configuration of the MISS, including the relevant surface orientations and incident angles, is shown in Fig. S14. By integrating this geometric configuration with Eq. (1), a basic fitting model based solely on device geometry for the angle was derived, with the specific derivation details in Section S6 of the Supplementary Information. The incident angles are fitted based on the experimental data in Fig. S15a. According to Eq. (S28), the fitting results for the basic fitting model are shown in Fig. S15b. There are significant discrepancies from the ideal, indicating that additional physical mechanisms were not accounted for, and a more comprehensive fitting model needs to be developed.

The discrepancies may be attributed to factors such as the lateral drift of charge carriers^36,37^ and increased optical loss from the longer optical path length of obliquely incident light^38^. If the direction of light is represented by the unit vector $\vec{L}$, and the normal vectors of the surfaces of detector A, B, and C are represented by ${\vec{n}}_{A}$, ${\vec{n}}_{B}$, and ${\vec{n}}_{C}$, respectively, the loss associated with the deviation between the incident light direction and the surface normal can be quantified as ($1-{\mathop{n}\limits^{ \rightharpoonup }}_{i}\cdot \mathop{L}\limits^{ \rightharpoonup }$). The expression ${e}^{-\beta (1-{\mathop{n}\limits^{ \rightharpoonup }}_{i}\cdot \mathop{L}\limits^{ \rightharpoonup })}$ represents the loss rate^39^, where *β* is an empirically determined parameter. Furthermore, the surface area of detector B is significantly larger than the inclined detetors and generates significantly more photogenerated carriers. The isolation trenches prevent some of the drift, but some carriers are still able to drift toward the two adjacent inclined detectors. The equation ${I}_{{drift}}=\eta {I}_{{pho}}\sqrt{1-{\left({\mathop{n}\limits^{ \rightharpoonup }}_{i}\cdot \mathop{L}\limits^{ \rightharpoonup }\right)}^{2}}$ represents the lateral drift current of the PV cell^40^, where $\eta$ is the lateral drift efficiency factor and ${I}_{{pho}}$ is photocurrent. The lateral drift efficiency is defined as the ratio between the drift current of photogenerated carriers due to the lateral electric field or concentration gradient and the original photocurrent. By incorporating the effects of losses and the lateral drift current, the output of the three detectors can be derived from Eq. (1) as follows:

2 $${I}_{A}=\kappa {{\rm{S}}}_{0}Esin(\theta -\alpha )\cdot {e}^{-\beta [1-sin\left(\theta -\alpha \right)]}(1+\eta cos\theta )$$

3 $${I}_{B}=\kappa {{\rm{S}}}_{1}Esin\theta \cdot {e}^{-\beta (1-sin\theta )}(1-2\eta cos\theta )$$

4 $${I}_{C}=\kappa {{\rm{S}}}_{0}Esin(\theta +\alpha )\cdot {e}^{-\beta [1-sin\left(\theta +\alpha \right)]}(1+\eta cos\theta )$$

where *S*_*0*_ is the illuminated surface area of detectors A and C, and *S*_*1*_ is the illuminated surface area of detector B.

To simplify calculations, a Taylor series expansion was applied to Eqs. (2)–(4). The resulting expanded forms were substituted into the expression for *D*, yielding an intensity-independent equation to further solve for the angle:

5 $$D=\frac{{I}_{C}-{I}_{A}}{{I}_{B}}\approx \frac{2{{\rm{S}}}_{0}\beta \left(1+\eta cos\theta \right)cos\theta sin\alpha }{{{\rm{S}}}_{1}sin\theta (1-\beta -\beta sin\theta )(1-2\eta cos\theta )}$$

Directly computing the angle using Eq. (5) is still relatively complex. However, the problem can be effectively solved by employing the Newton-Raphson iteration method in MATLAB^41^. The final equation for the solar angle is as follows:

6 $$\theta =\arctan \left[\frac{2{{\rm{S}}}_{0}\beta \eta sin\alpha }{{{\rm{S}}}_{1}D\left(1-\beta \right)}\right]+\frac{2\eta sin\alpha }{1-\beta }$$

Equation (6) represents the functional relationship between the angle and the generated photocurrents of the three detectors, accounting for possible losses. Furthermore, parameters $\beta$ and $\eta$ can be solved by analyzing the detector’s behavior under very small angles ($\theta \to 0^\circ$). When $\theta$ approaches $0^\circ$, the approximations $\sin (\theta )=0$ and $\cos (\theta )=1$ can be made. By setting ${G}_{1}=\frac{{I}_{A}}{{I}_{B}}$ and ${G}_{2}=\frac{{I}_{C}}{{I}_{B}}$, the following equations are obtained:

7 $${G}_{1}=\frac{{{\rm{S}}}_{0}(cos\alpha -sin\alpha )(1-\beta cos\alpha )(1+\eta cos\theta )}{{{\rm{S}}}_{1}(1-sin\theta )(1-2\eta cos\theta )}$$

8 $${G}_{2}=\frac{{{\rm{S}}}_{0}(cos\alpha +sin\alpha )(1+\beta cos\alpha )(1+\eta cos\theta )}{{{\rm{S}}}_{1}(1-sin\theta )(1-2\eta cos\theta )}$$

Combining with Eq. (7) and Eq. (8), the solution is obtained:

9 $$\beta =\frac{{G}_{1}(cos\alpha +sin\alpha )-{G}_{2}(cos\alpha -sin\alpha )}{[{G}_{1}(cos\alpha +sin\alpha )+{G}_{2}\left(cos\alpha -sin\alpha \right)]cos\alpha }$$

10 $$\eta =\frac{{{\rm{S}}}_{1}[{G}_{1}(cos\alpha +sin\alpha )+{G}_{2}\left(cos\alpha -sin\alpha \right)]-{{\rm{S}}}_{0}cos2\alpha }{2{{\rm{S}}}_{1}[{G}_{1}(cos\alpha +sin\alpha )+{G}_{2}\left(cos\alpha -sin\alpha \right)]+{{\rm{S}}}_{0}cos2\alpha }$$

Generally, the value of $\beta$ is on the magnitude of 10^−^^2^, while $\eta$ is between 0 and 0.3. Consequently, the effect of optical path loss on the measurement results of the device is negligible, and the primary difference of the comprehensive model arises from the lateral drift of carriers. Using the new equations, a new comprehensive model was created, and the incident angle was fitted from the output currents of the three detectors. Figure S15c presents the fitting results based on the new model, demonstrating the model’s applicability for angle determination within the range [15°, 165°], which corresponds to a FOV of ±75°.

**Fig. 5:**
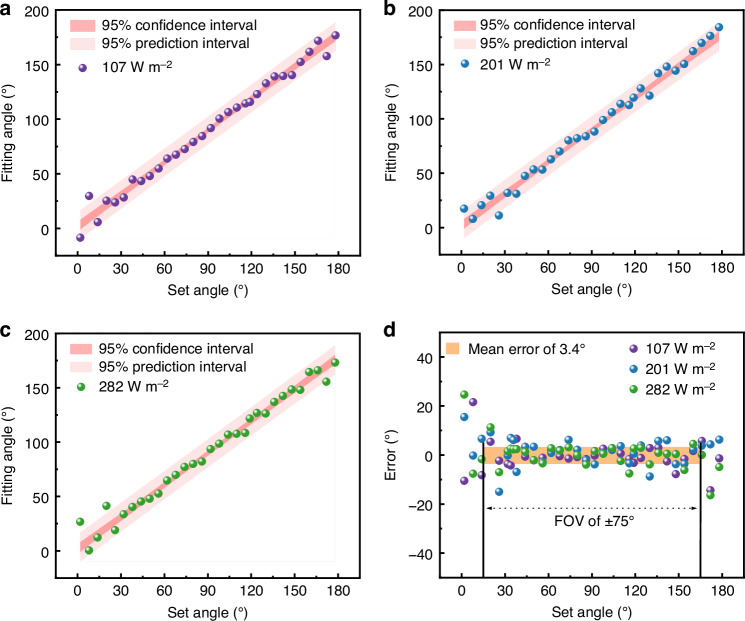
Solar angle fitting model results. **a**–**c** The fitted angle at various intensities. **d** The deviation between the fitted angle and the set angle

To further confirm the universal applicability of this model and its independence from intensity, the model was validated using data from a different chip, not only covering the same range of angles but also under varying intensities (Fig. 5a–c). The results confirm the independence of angular determination from intensity, as the model exhibited consistent agreement across all tested intensities. The confidence and prediction interval for the fit were also calculated to determine the accuracy of the model’s mean prediction and the expected range for a new single observation, respectively. When the light is shown vertically onto the chip, the confidence interval has a maximum range of ±2.2° across the three intensities. The error is higher at the extremes, as light with high or low angles is partially blocked by the device or its packaging, but the confidence interval is still within ±3.6° showing that the fit is still able to guide some estimates. The prediction interval is higher than the confidence interval at ±12.1°, which indicates that while the model itself is well-defined and accurate on average, the precision of any single prediction is currently limited by the inherent noise in the data.

To verify the accuracy of the MISS, the deviation was calculated by subtracting the set angle from the fitted angle calculated by the comprehensive model (Fig. 5d). The result illustrates that the detectors and fitting model combined can effectively measure the angle of incident light in an unprecedented FOV of ±75° with a mean angular error of 3.4° across all tested intensities. Detailed calculation process and raw data for this calculation can be found in Section S9 of the Supplementary Information. A performance comparison with other reported single-chip solar angle sensors is provided in Table 1. In general, most reported solar angle sensors can measure angles within a FOV of ±55° with an error below 5°. In contrast, the MISS has a larger FOV while maintaining the higher accuracy. This larger measurement range enables the sensor chip to cover the full range of rotation of a PV panel without requiring a dedicated rotating platform, unlike many other sensors with smaller FOVs.

**Table 1:** Comparison of the solar angle measurement performance

Sensor Module	FOV	Mean error	Reference
MISS	±75°	3.4°	This work
SiC image sensor	±37°	5.7°	^24^
PV sub-module	±62.8°	5°	^22^
Light source tracking	±45°	5°	^42^
Micromachined vector sensor	±45°	1°	^43^
Ultraviolet (UV) sensor	±55°	-	^25^
Irradiation angle sensor	±48°	0.3°	^26^
Dual-axis sun tracker	±30°	-	^44^
Direction tracking system	±45°	1.8°	^15^
Pyramidal sun sensor	±60°	7.8°	^45^

### Intensity fitting analysis

The relationship between the signal generated by the detector and the incident light intensity is analyzed through a linear fitting model to allow the quantitative evaluation of the intensity based on the detector response output. The correlation between the intensity and the response of the detector was demonstrated in the previous section. Since the optical path loss on the detector is negligible, the relationship between photocurrent response and intensity can be given by a simplified form of Eqs. (2)–(4) from the previous analysis:

11 $${I}_{A}=\kappa {{\rm{S}}}_{0}Esin(\theta -\alpha )(1+\eta cos\theta )$$

12 $${I}_{B}=\kappa {{\rm{S}}}_{1}Esin\theta (1-2\eta cos\theta )$$

13 $${I}_{C}=\kappa {{\rm{S}}}_{0}Esin(\theta +\alpha )(1+\eta cos\theta )$$

Figures 4e, f and S13 represent the change in detector response to changes in intensity at a specific solar angle. Equations (11)–(13) can be rewritten to determine the equations of ${k}_{A}$, ${k}_{B}$, and ${k}_{C}$:

14 $${k}_{A}=\kappa {S}_{0}sin\left(\theta -\alpha \right)\left(1+\eta cos\theta \right)$$

15 $${k}_{B}=\kappa {S}_{1}sin\theta \left(1-2\eta cos\theta \right)$$

16 $${k}_{C}=\kappa {S}_{0}sin\left(\theta +\alpha \right)\left(1+\eta cos\theta \right)$$

where ${k}_{A}$, ${k}_{B}$ and ${k}_{C}$ represent the ratios of the generated photocurrent to intensity for detectors A, B, and C, respectively. For a given angle, coefficients ${k}_{A}$, ${k}_{B}$ and ${k}_{C}$ are fixed, establishing a linear dependence of the photocurrent *I* on the irradiance *E*, as confirmed by Figs. 4e, f and S13.

Because these coefficients vary with angle, the solar angle determined by the MISS device allows the values of ${k}_{A}$, ${k}_{B}$, and ${k}_{c}$ to be interpolated for any angle within the device’s FOV. Theoretically, three current-intensity relationships ($E=\frac{{I}_{A}}{{k}_{A}}$, $E=\frac{{I}_{B}}{{k}_{B}}$, and $E=\frac{{I}_{c}}{{k}_{c}}$) can be derived from Eqs. (11)–(13), respectively. The linear relationship is used to obtain the intensity from the output of the three separate detectors, which was then averaged to obtain a more accurate intensity measurement. Therefore, the expression of the intensity is further obtained:

17 $$E=\frac{1}{3}(\frac{{I}_{A}}{{k}_{A}}+\frac{{I}_{B}}{{k}_{B}}+\frac{{I}_{c}}{{k}_{c}})$$

**Fig. 6:**
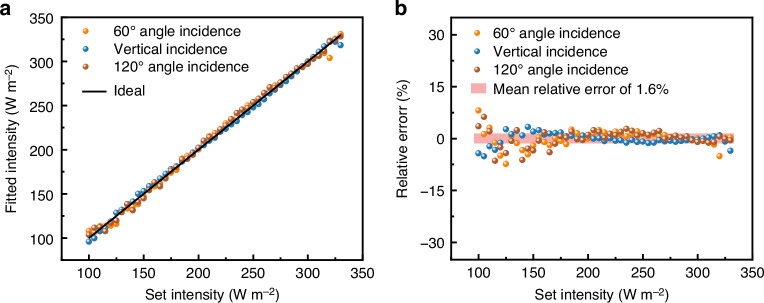
Intensity fitting model results. **a** The fitted intensity at various incident angles. **b** The relative error between the fitted intensity and the actual intensity

Figure 6a shows the fitting intensity under the incident light at angles of 60°, 90°, and 120°. The fitting results at angles of 60°, 90°, and 120° reveal that the fitted intensity at the three incident angles is predominantly concentrated around the ideal reference line, demonstrating the model’s reliability in predicting intensity. Experimental validation of the model is performed using the mean relative error. Further, the relative error, defined as the ratio of the difference between the measured value and the true value to the true value, is calculated to reflect the accuracy of the fitting model. The raw data and mean error calculations to validate the fitting model can be found in the newly added Section S9 of the Supplementary Materials. Figure 6b exhibits a mean relative error of 1.6% over the range of tested intensities. It is worth noting that as the input intensity increases, the mean relative error decreases, as the absolute error value is similar across the tested intensities. This means that for nominal sunlight intensity, which is generally estimated at 1000 W/m^2^, the relative error in intensity should be even lower, and this level of accuracy is sufficient for photovoltaic optimization in PV systems^16^. A performance comparison with other reported methods is provided in Table 2. While the mean error for most existing sensors in the literature is within 5%, the MISS achieves a significantly smaller mean error.

**Table 2:** Comparison of the intensity measurement performance

Sensor Module	Mean error	Reference
MISS	1.6%	This work
LED sensor	2.4%	^46^
LSIF	10%	^18^
PIRATA measurement	8%	^47^
SORCE	5%	^48^
General empirical model	1–2%	^49^
PSR	1.7–2%	^50^

## Conclusion

This work presents a multifunctional device that simultaneously measures solar angle and intensity on a single chip with a compact footprint of 9 mm × 9 mm. The tradeoff between FOV and accuracy in miniaturized solar sensors is addressed by integrating three detectors with differently angled surfaces to increase the FOV to an unprecedented ±75°. High accuracy with a mean angular error of 3.4° and a mean relative intensity error below 1.6% was achieved using physics-based fitting models that account for nonidealities such as lateral carrier drift and empirically determined losses with minimal computational overhead. As a result, the MISS fills an essential role in real-time control of solar tracking, MPPT, and array reconfiguration in photovoltaic systems. Beyond photovoltaic applications, its compact and integrated design offers broad potential for space-based solar monitoring and distributed environmental sensing.

While the current work demonstrates strong potential, further device optimization could improve performance by enhancing current output and lateral carrier drift suppression. For example, increasing the ion implantation dose or reducing reflections from various surfaces on the sensor chip may enhance photocurrents, allowing further improvements in signal-to-noise ratio and accuracy. Partially shielding the light-receiving regions and using deeper isolation trenches to reduce the generation and drift of carriers formed in the areas adjacent to the detector can similarly improve accuracy. These optimizations also enhance the prediction interval’s accuracy across its entire range, with the most significant improvements occurring at extreme angles. Additional functionality can be achieved by expanding the surface area of the detector to provide both sensor signals and power for a microsystem. Additionally, the functionality of the device can be further improved by expanding the solar angle measurements from a single-axis measurement to a dual-axis measurement by leveraging the symmetric multi-detector outputs.

## Supplementary information

Supplementary Information
