# Supplementary material for: Wide field of view multifunctional solar sensor for photovoltaic power management via measurement of solar angle and intensity
Source: Microsyst Nanoeng. 2026 Feb 11;12:59. doi: 10.1038/s41378-025-01154-4 (PMC12894965; doi:10.1038/s41378-025-01154-4)
Supplement: Supplementary file 1 — Supplementary Information [file 41378_2025_1154_MOESM1_ESM.pdf]

Supplementary Information

Wide Field of View Multifunctional Solar Sensor for Photovoltaic Power Management via Accurate Measurement of Solar Angle and Intensity

Yifeng Liu[1], Qingfeng Wu[1], Haizhao Feng[1], Yier Xia[1], Minghao Xu[1], Xiangyu Zhao[1,*], Philippe Basset[2], and Xiaohong Wang[1,*]

[1]School of Integrated Circuit, Tsinghua University, Beijing 100084, China

[2]Univ Gustave Eiffel, CNRS, ESYCOM, F-77454 Marne-la-Vallée, France

*Corresponding authors

These authors contributed equally: Yifeng Liu, Qingfeng Wu.

Email: wxh-ime@tsinghua.edu.cn (Xiaohong Wang), xy_zhao@tsinghua.edu.cn (Xiangyu Zhao)

S1. The characteristics of the solar cell

The solar cell various characteristics are defined as[1-3]:

$$I_{SC} = I_0 \left[\exp\left(\frac{qv}{nKT} - 1 \right) - I_{ph} \right] \tag{S1}$$

$$V_{oc} = \frac{KT}{q} \ln\left(1 + \frac{I_{sc}}{I_0} \right) \tag{S2}$$

$$FF = \frac{P_{max}}{I_{sc}V_{oc}} = \frac{I_{mp}V_{mp}}{I_{sc}V_{oc}} \tag{S3}$$

The fill factor (FF) formula given by Green[4,5]:

$$FF = \frac{v_{oc} - \ln(v_{oc} + 0.72)}{v_{oc} + 1} \tag{S4}$$

where, $v_{oc} = \frac{V_{oc}}{V_{th}}$ is defined as normalized V_{oc}.

$$\eta = \frac{P_{max}}{P_{in}} = \frac{I_{mp}V_{mp}}{P_{in}} = \frac{FFI_{sc}V_{oc}}{P_{in}} \tag{S5}$$

where I_{sc} is the short-circuit current density, I_0 is saturation current density, I_{ph} is photocurrent density, V_{oc} is the open-circuit voltage. P_{max} is the maximum power output that occurs at (V_{mp}, I_{mp}). P_{in} is the incident light power onto the cell.

S2. The IBC solar cell simulation model

In this simulation, the structure of an interdigitated back-contact (IBC) solar cell is constructed and calibrated on the previous experimental models by Silvaco TCAD tools. For simulations of the IBC solar cell using ATLAS Silvaco, the simulation model may involve Poisson's equations, Maxwell's equations, and carrier transport equations in 2D.

In this simulation, the calculation was based on Poisson's equation[6,7]:

$$\nabla \cdot E = \frac{\rho}{\varepsilon} = \frac{q}{\varepsilon}[p(x) - n(x) + N_D^+(x) - N_A^-(x)] \tag{S6}$$

where E is the electric field, ρ is the total charge, ε is the permittivity of the material, q is the

electric charge, p and n are the number of free holes and free electrons, respectively. N_D^+ and N_A^- are the ionized donor density and acceptor density with the position vector x, respectively.

The Galerkin finite element method (FEM) is used to solve Maxwell's equations based on the nodal discontinuous[8]. All four of Maxwell's equations are given by:

$$\nabla \times H = \frac{\partial D}{\partial t} + I \tag{S7}$$

$$\nabla \times E = -\frac{\partial B}{\partial t} + I_m \tag{S8}$$

$$D = \varepsilon E \tag{S9}$$

$$B = \mu H \tag{S10}$$

where D is the electric flux intensity, I is the current density, t is the time, H is the magnetic field, I_m is the magnetic density, E is the electric field, B is the magnetic flux intensity, and μ is the complex permeability of the material.

In semiconductor device modeling, there are three important processes that have been taken into account in the calculation: carrier generation, carrier transport, and recombination. The transport equations can be expressed as[6,9]:

$$I_n(x) = q\mu_n E(x) + qD_n \nabla_n(x) \tag{S11}$$

$$I_p(x) = q\mu_p E(x) + qD_p \nabla_p(x) \tag{S12}$$

Finally, the continuity equation for the electron can be formulated as[10]:

$$\nabla \cdot I_n(x) = q(G_n - U_n) \tag{S13}$$

$$\nabla \cdot I_p(x) = q\left(G_p - U_p\right) \tag{S14}$$

Eq. (S13) describes the electrostatics and electrodynamics of semiconductor materials. Where

U represents the net recombination rate and G represents the carrier generation of electrons and holes.

From the considered IBC model, the p-type Emitter is heavily doped where the minority carriers can be expressed as[11]:

$$I_n = q\mu_n nE + qD_n \frac{dn}{dx} \quad \text{(S15)}$$

$$I_p = q\mu_p nE + qD_p \frac{dp}{dx} \quad \text{(S16)}$$

$$\frac{1}{q}\frac{dI_n}{dx} = G - \frac{1}{\tau_n}(p - p_0) \quad \text{(S17)}$$

$$\frac{1}{q}\frac{dI_p}{dx} = G - \frac{1}{\tau_p}(p - p_0) \quad \text{(S18)}$$

where I_n and I_p are the electron and hole current densities, μ_n and μ_p are the electron and hole mobilities, E is the electric field, q is Coulomb charge, ∇_n and ∇_p are the electron and hole concentration gradients, and finally, D_n and D_p are the electron and hole diffusion coefficients, respectively. p_0 is the minority carrier equilibrium concentration, n and p are the minority carrier concentrations, and τ_n is the minority carrier lifetime.

S3. Structural parameters determination by Quokka simulation

Quokka (obtain the website of the simulator: https://github.com/quokkaproject/quokka) is used for the fast simulation of silicon solar cell devices in one to three dimensions. It employs simplifications to the general semiconductor carrier transport model, resulting in much less computational effort. Thus, Quokka enables to simulate moderately complex 3D silicon solar cell device geometries in short computation times on standard computers, while providing a similar

level of accuracy and generality. Herein, some ordinary simulations have been made from three aspects: (1) Determine the optimal silicon wafer thickness. (2) Understand the influence of p-n junction depth. (3) Clarify the influence of p-n junction width.

The Quokka simulator is used to perform a simple structural simulation of solar cell[12], with the simulation model of the solar cell illustrated in **Fig. S1**. Generally, a thin wafer is not conducive to the absorption of solar light, while a thick wafer leads to increased carrier recombination[13,14]. Given this cause, the Quokka2.2.5 simulator could be used to comprehend the effect of the wafer thickness on the conversion efficiency of solar cell. The simulation result indicates that the solar cell possesses the maximum photoelectric conversion efficiency (up to 23.75%) when the thickness of the wafer reaches 350 μm in Fig. **S4**a. Due to the presence of KOH etching area in the actual device, a 400 μm wafer will be selected in the end.

Moreover, the size of the p-n junction likewise impacts the conversion efficiency of solar cell. The junction depth of the p-n junction has been set to 0.3-1.5 μm, respectively. Based on the simulation results in **Fig. S4**b, whether it is p junction or n junction, the conversion efficiency of solar cell can be only boosted by 0.004% as the junction depth increases from 0.3 to 1.5 μm. As a consequence, the design of the solar cell sensor can opportunely ignore the effect of junction depth. Subsequently, the effect of the junction width of the p-n junction has been investigated. The simulation result in **Fig. S4**c reveals that the conversion efficiency of the solar cell would decrease slightly with the increase of the n-junction width. This is mainly due to the fact that the increase in the n-type heavy doping region could increase the recombination of the carriers[15,16]. On the

contrary, the conversion efficiency would increase significantly with the increase in the p-junction width. This may be due to the increase in increasing width of the p-junction is conducive to the absorption of long-wave light[15,16]. Importantly, it is necessary to ensure that the total width of the p-n junction does not exceed the projection width (175 μm) of the inclined plane. Given the above analysis, the design of 10 μm wide n-junction and 110 μm p-junction are employed.

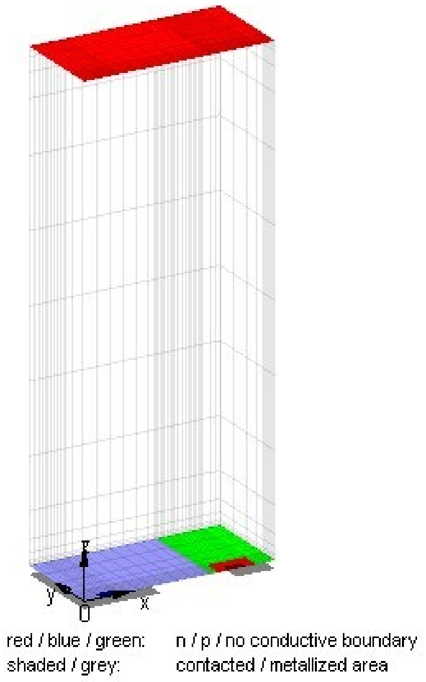

Fig. S1. The model used for simulation based on Quokka2.2.5 simulator.

S4. Structural parameters optimization by Silvaco TCAD simulation

Typically, photoelectric characteristics are optimized by tuning the structural parameters of p-n junction. The optical-electrical simulation module in the Silvaco TCAD 2020 simulation software (Silvaco, USA) is used for the simulation of the complex geometry of the device. For this work, simulations have been made from two aspects: (1) Common structural optimization is carried out by adjusting the depth of the isolation channel. (2) The ion implantation doses,

involving the front-surface field (FSF), the back-surface field (BSF) and emitter electrode, are swept, to optimize the photoelectric sensing characteristic. The specific simulation conditions and the specific parameters of the device are displayed in **Table S1** and **Table S2**, respectively.

Table S1. The specific simulation conditions.

Simulation condition	Simulation parameter
Wafer thickness	400 μm
Light intensity	1 W cm^{-2}
Spectrum	AM1.5
KOH etching depth	250 μm
DRIE etching depth	0-100 μm

Table S2 Key structural parameters of the MISS device

Structure	Width (μm)	Length (μm)	Thickness (μm)
n-type wafer	-	-	400
FSF	6000	6000	-
BSF	10	4900/2000	-
Emitter	110	4900/2000	-
Isolation channel	5	5000/2200	50
KOH etching	6000	6000	250
Metal electrode	20/120	4910/2010	0.2

The Fermi-Dirac model, Concentration-dependent mobility model (CONMOB), Shockley–Read–Hall (SRH), Auger, and Optical recombination (OPTR) are invoked in the simulation program. The rate of Auger recombination can be written as[17,18]:

$$R_{Auger} = \left[c_n(T_L)N + c_p(T_L)P\right](NP - N_0P_0) \tag{S19}$$

where $c_n(T_L)$ and $c_p(T_L)$ are the Auger coefficients for holes and electrons, respectively.

Shockley-Read-Hall Recombination is defined as:

$$R_{SRH} = \frac{pn - n_{ie}^2}{\tau_n\left[n + n_{ie}\exp\left(\frac{E_{TRAP}}{KT_L}\right)\right] + \tau_p\left[p + n_{ie}\exp\left(\frac{-E_{TRAP}}{KT_L}\right)\right]} \tag{S20}$$

In this work, we demonstrate the effect of doping concentration on the front surface field, back surface field, and emitter for a high-efficiency . The simulated structure consists of an n-type nitride silicon wafer of 400 μm thickness with a carrier lifetime peak of 1 msec. To speed up the simulation process, the original structure has been simplified (**Fig. S2**), primarily ensuring that the projected area of the bottom sensor B is consistent with the projected areas of the two inclined surfaces.

The n^+ front diffusion provides the front-surface field (FSF), to reduce the front surface recombination and to improve selectivity in carrier collection. The FSF layer plays a pivotal role in mitigating surface recombination while simultaneously enhancing the selectivity of carrier collection in the solar cell[19,20]. **Fig. S5**a illustrates the relationship between the maximum power point (P_{max}) and the conversion efficiency with the phosphorus ion implantation dose for FSF. From the analysis, there is a gradual increasing phenomenon in both the maximum power point and conversion efficiency within the implantation dose range of 5×10^{10} cm^{-2} to 2×10^{12} cm^{-2},

followed by a dramatic decrease after 3×10^{13} cm^{-2}. This phenomenon indicates that the optimal ion implantation dose is 2×10^{12} cm^{-2} for FSF.

When the back surface of the solar cell is subjected to a sufficiently high voltage bias, the rear Emitter electrode will immediately inject minority carriers into the n-type silicon region[21,22]. To attain a high injection efficiency, it is also important to precisely control over the ion implantation dose of the Emitter electrode. Taking sensor A as a case, the ion implantation dose of the Emitter electrode has been thoroughly simulated. The simulation results in **Fig. S5**b reveal that with an increase in the implantation dose, both the P_{max} and conversion efficiency of Sensor A also exhibit a significant enhancement. However, once the implantation dose surpasses 2×10^{12} cm^{-2}, the growth rate begins to decelerate. This may be attributed to an increase in Emitter electrode recombination velocity, resulting in a significant reduction in injection efficiency[19,23]. Considering the process cost, the optimal implantation dose for the p^+ Emitter is approximately 4×10^{15} cm^{-2}.

It is widely recognized that the BSF enhances the performance of solar cell by reducing the surface recombination rate[22,24], typically achieved by high doping concentrationsin the fabrication process. **Fig. S5**c displays the functional relationship between P_{max} and conversion efficiency with the implantation dose of the BSF layer. The result illustrates that there is a limit when the implantation dose of BSF is below 10^{16} cm^{-2}. This can be attributed to the failure to form a sufficiently strong built-in electric field, making it difficult for the photogenerated carriers generated on the front surface to be collected effectively[25,26]. However, at extremely high implantation doses, Auger recombination at the back surface occurs, leading to a slowdowninthe improvement of conversion efficiency. Thus, the optimal ion implantation dose is approximately

3×10^{16} cm^{-2} for BSF.

The appropriate isolation channel plays a pivotal role in preventing the current drift between the three detectors[14,27]. In silicon-based solar cell, the breakdown voltage of the isolation channel of the air medium can be expressed in Eq.(S21)[28]:

$$V_{BD} = E_{BD} \times d \tag{S21}$$

where V_{BD} is the breakdown voltage (V), E_{BD} is the breakdown field strength of the medium (V m^{-1}), d is the isolation channel width (m). The width of the designed isolation channel is 5 μm. Typically, the breakdown field strength of air is 3×10^{6} V m^{-1}. In this case, the designed isolation channel has a breakdown voltage up to 15 V. Moreover, the open-circuit voltage of solar cell is 0.5-0.8 V[29-31]. Hence, the 5 μm wide isolation channel is sufficient to eliminate the interference between the three sensors. Besides, the appropriate depth of the isolation channel can effectively insulate the longitudinal drift of the current between the various sensors[28,32]. Therewith, an insightful analysis of the effect of the depth of the isolation channel has been carried out. **Fig. S6** explicates that as the depth of the isolation tank increases to 70 μm, the blocking effect of the isolation channel becomes increasingly obvious. Further, when the depth of the isolation channel exceeds 70 μm, it completely suppresses the current drift between the various detectors.

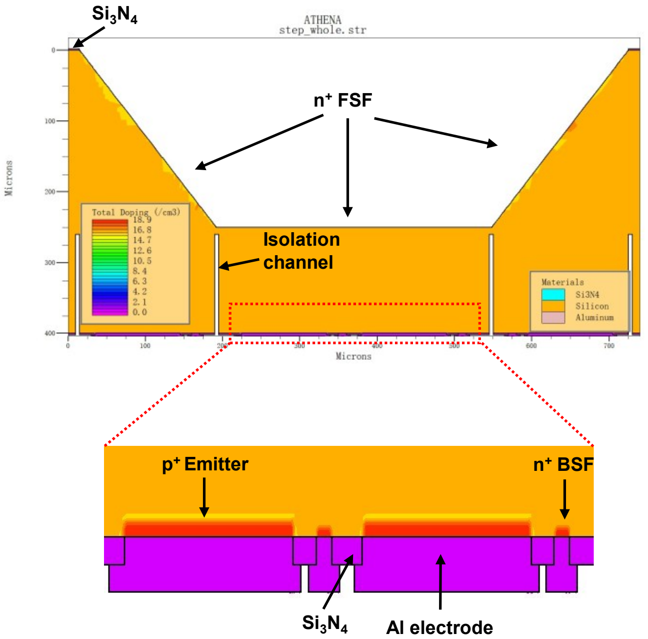

Fig. S2. The model used for simulation based on Silvaco TCAD 2020 simulation software.

S5. Bigaussian interpolation process

Bigraussian fitting is a mathematical method used to analyze data with two peaks or an asymmetric distribution. It precisely describes complex signals by superimposing two Gaussian functions. When $x < \mu$, its mathematical model is:

$$y = y_0 + He^{-0.5\left(\frac{x-\mu}{\sigma_1}\right)^2} \tag{S22}$$

else

$$y = y_0 + He^{-0.5\left(\frac{x-\mu}{\sigma_2}\right)^2} \tag{S23}$$

where y_0, H, and μ are regarded as baseline offset value, the peak height value, and the peak mean value, respectively. Additionally, σ_1 and σ_2 are defined as the standard deviations on either side of the peak.

The implementation of Bigaussian fitting requires a systematic flow: (1) Firstly, through

visual inspection or derivative analysis, the number and positions of the peaks are preliminarily determined; (2) Then, the non-linear least squares algorithm (such as Levenberg-Marquardt) is used for parameter optimization. This process requires careful setting of initial values to avoid getting stuck in local optimal solutions; (3) Finally, the fitting quality is evaluated through residual analysis, R^2 fitting goodness, and information criteria, etc. Compared with single Gaussian fitting, this method has the advantage of being able to more accurately describe complex peak shapes, but it also faces challenges such as strong parameter correlation and difficult convergence. Usually, it is necessary to combine physical constraints or regularization methods to improve the fitting stability. The detailed fitting parameters for **Fig. 4**a-c are displayed in **Table S3**.

Table S3. The detailed fitting parameters about Bigraussian fitting.

Detector	Intensity (W m^{-2})	y_0	H	μ	σ_1	σ_2
A	107	-0.0014	0.023	113.65	47.521	31.406
	201	-0.0026	0.046	111.61	40.459	31.903
	282	-0.0108	0.073	115.89	51.651	32.014
B	107	-0.0317	0.144	86.54	43.362	48.697
	201	-0.0479	0.287	86.09	40.791	46.368
	282	-0.0582	0.429	85.61	40.005	46.005
C	107	0.00134	0.017	70.71	30.748	32.691
	201	0.00067	0.034	65.97	24.716	35.151

282	0.00029	0.053	65.26	25.708	34.855

S6. Derivation of a basic model for the angle

The effective intensity of the incident light can be calculated by multiplying the dot product between the normal unit vector of the illuminated surface and the vector representing illumination. Based on this, a basic physical model for the MISS device is established in **Fig.** S15. Hypothetically, the direction of light is represented by the unit vector $\vec{L}$. Furthermore, the normal vectors of the surfaces of detectors A, B, and C are represented by $\vec{n}_A$, $\vec{n}_B$, and $\vec{n}_C$, respectively. According to the principle outlined in Eq. (1), the output of detectors A, B and C can be expressed as:

$$I_A = \kappa_A S_0 E \cdot (\vec{n}_A \cdot \vec{L}) = \kappa_A S_0 E sin(\theta - \alpha) \quad \text{(S24)}$$

$$I_B = \kappa_B S_1 E \cdot (\vec{n}_B \cdot \vec{L}) = \kappa_B S_1 E sin\theta \quad \text{(S25)}$$

$$I_C = \kappa_C S_0 E \cdot (\vec{n}_C \cdot \vec{L}) = \kappa_C S_0 E sin(\theta + \alpha) \quad \text{(S26)}$$

where S_0 is the surface area of the inclined detectors A and C, S_1 is the surface area of detector, α is the inclination angle of the KOH etched planes of detectors A and C and has the value of 54.7°. Theoretically, detector A begins to generate a lower output at incident light angles smaller than α, while detector C generates lower outputs within the range of [180°-α, 180°]. Furthermore, since MISS is a single chip device with the identical materials and environments of detectors A, B, and C, it can be assumed that $\kappa_A = \kappa_B = \kappa_C$. Therefore, the ratio between detector outputs *D* can be simplified as:

$$D = \frac{I_C - I_A}{I_B} = \frac{2S_0 sin\alpha}{S_1 tan\theta} \tag{S27}$$

Solving for the angle of incident light based on the detector, the following equation is obtained:

$$\theta = \arctan\left[\frac{2S_0 sin\alpha}{S_1 D}\right] \tag{S28}$$

A basic fitting model can be generated with Eq. (S28) with the areas of S_0 and S_1 set to 1.496 mm^2 and 35.283 mm^2, respectively.

S7. Taylor expansion estimation

The basic form of Taylor expansion is:

$$f(x) = \sum_{i=0}^{n} \frac{f^i(x_0)}{i!}(x - x_0)^i + R_i(x) \tag{S29}$$

As a result, the exponential part in Eq. (8-10) can be approximately expressed as:

$$e^{-\beta[1-sin(\theta-\alpha)]} = 1 - \beta[1 - sin(\theta - \alpha)] \tag{S30}$$

$$e^{-\beta(1-sin\theta)} = 1 - \beta(1 - sin\theta) \tag{S31}$$

$$e^{-\beta[1-sin(\theta+\alpha)]} = 1 - \beta[1 - sin(\theta + \alpha)] \tag{S32}$$

Therefore:

$$I_A = \kappa S_0 E sin(\theta - \alpha)(1 + \eta cos\theta)\{1 - \beta[1 - sin(\theta - \alpha)]\} \tag{S33}$$

$$I_B = \kappa S_1 E sin\theta(1 - 2\eta cos\theta)\{1 - \beta(1 - sin\theta)\} \tag{S34}$$

$$I_C = \kappa S_0 E sin(\theta + \alpha)(1 + \eta cos\theta)\{1 - \beta[1 - sin(\theta + \alpha)]\} \tag{S35}$$

S8. Calculation of the confidence and predition intervals

The confidence interval (*CI*), which provides the estimated range of the mean value, and the prediction interval (*PI*), which provides an estimated range for the value, are calculated using:

$$CI = \hat{y}_h \pm t_{(1-\alpha/2, n-2)} s \sqrt{\frac{1}{n} + \frac{(x_0 - \bar{x})^2}{\sum (x_i - \bar{x})^2}} \tag{S36}$$

$$PI = \hat{y}_h \pm t_{(1-\alpha/2, n-2)} s \sqrt{1 + \frac{1}{n} + \frac{(x_0 - \bar{x})^2}{\sum (x_i - \bar{x})^2}} \tag{S37}$$

The $\hat{y}_h$ is the estimated mean, $t_{(1-\alpha/2,n-2)}$ is the t-distribution value at a specified confidence level and degree of freedom, s is the square root of the variance of the error term, n is the number of samples, x_0 is the location of the prediction, $\bar{x}$ is the sample mean of x, and $\Sigma(x_i - \bar{x})^2$ is the sum of the squares of the difference between each x and the mean x value. In our case specifically, the confidence interval shows how well the model determines the average relationship between the detector outputs and the solar angle while the prediction interval tells us the expected range for a single angle or intensity measurement calculated by the fitting model.

S9. Detailed calculation process for the mean error

The raw data of detector output at different angles and intensities can be found in **Table S4** and **Table S5**. These raw values are used to plot the relationship curves of current-angle and current-intensity (**Fig. 4** and **Fig. S13**). To make the fitting process easier, a fixed interval (whether for angle or intensity) is needed and thus it is necessary to resample the data using interpolation. For the angle, Bigaussian interpolation method is employed (the fitting process is shown in Section S5). For the intensity, a simple linear interpolation is employed. After resampling the data, new angle-current or intensity-current relationship will be obtained. Then the set angle/intensity and their corresponding current are extracted on the new current-angle and current-intensity

relationship curves. The interpolatedinterpolated angle-current and intensity-current data are shown in **Table S6** and **Table S7**.

The interpolated datainterpolated and the fitting model (including the angle fitting model and the intensity fitting model) are inputted into MATLAB to obtain the fitting angle and intensity values. Its MATLAB code is as follows:

```
a = [data from Detector A]

b = [data from Detector B]

c = [data from Detector C]

r = [list of the angle or intensity data]

X = [fitting model]

coeff = linsolve (X, r)

calculated_data = X*coeff
```

The error is analyzed based on the fitting data and the set interpolated data. For angle error analysis, subtract the corresponding set interpolated angle data from the fitted angle data and extract the difference. Then, take the absolute value of the difference to ultimately obtain the error result of the angle. Finally, take the average of all error results to obtain the mean angular error. The error formula is as follows:

$$Error_{angle} = \frac{\sum_{i=angle} ABS(\text{angle} - \text{calculated angle})}{x_i}$$

Similarly, for intensity error analysis, subtract the corresponding set interpolated intensity data from the fitting intensity data and extract the difference. Then, take the absolute value of the difference to ultimately obtain the error result of the intensity. Divide the error result by the

corresponding intensity value and take the percentage to obtain the mean relative intensity error.

The error formula is as follows:

$$Error_{intensity} = \frac{\sum_{i=intensity} ABS(\frac{\text{intensity} - \text{calculated intensity}}{\text{interpolated intensity}})}{x_i}$$

The specific fitting data and error analysis are shown in **Table S8** and **Table S9**.

Table S4. The testing data for current-angle relationship.

Intensity: 107 W m^{-2}						Intensity: 201 W m^{-2}						Intensity: 282 W m^{-2}					
Angle (°)	I_A (μA)	Angle (°)	I_B (μA)	Angle (°)	I_C (μA)	Angle (°)	I_A (μA)	Angle (°)	I_B (μA)	Angle (°)	I_C (μA)	Angle (°)	I_A (μA)	Angl (°)	I_B (μA)	Angl (°)	I_C (μA)
173.3	0.002	173.8	0.002	172.4	0.003	171.6	0.003	176.0	0.002	177.3	0.006	182.5	0.002	173.4	0.002	174.1	0.002
164.4	0.004	162.0	0.010	166.7	0.009	165.6	0.008	167.4	0.013	169.8	0.023	167.6	0.002	164.2	0.002	163.7	0.002
157.8	0.008	155.3	0.016	158.6	0.020	156.7	0.016	160.6	0.025	161.2	0.048	159.6	0.002	156.5	0.002	152.0	0.002
144.7	0.014	145.9	0.024	149.0	0.033	150.4	0.025	155.1	0.042	155	0.071	146.5	0.002	146.9	0.003	141.3	0.004
137.7	0.016	139.0	0.031	141.6	0.044	143.7	0.040	149.9	0.060	147.2	0.110	136.1	0.003	136.5	0.004	131.7	0.009
127.6	0.020	133.5	0.035	136.0	0.049	136.2	0.054	144.0	0.083	136.9	0.172	128.8	0.005	128.3	0.008	123.2	0.013
116.0	0.022	123.3	0.040	126.0	0.058	131.0	0.063	138.5	0.102	131.5	0.205	117.6	0.008	120.8	0.011	116.5	0.019
108.9	0.021	115.3	0.042	120.4	0.061	125.2	0.075	130.2	0.138	124.2	0.253	109.3	0.010	113.5	0.015	109.5	0.025
100.2	0.020	109.5	0.043	112.2	0.062	119.7	0.084	123.9	0.166	114.7	0.308	102.7	0.012	104.7	0.019	103.8	0.030
94.1	0.020	102.0	0.041	103.2	0.058	113.7	0.095	116.6	0.190	104.8	0.346	95.0	0.015	99.1	0.022	96.9	0.034
85.6	0.019	94.1	0.039	96.4	0.057	108.7	0.102	108.8	0.213	99.3	0.355	85.6	0.017	93.0	0.026	88.3	0.042
76.1	0.017	88.1	0.037	89.1	0.053	100.4	0.108	101.4	0.228	93.2	0.360	76.3	0.018	87.0	0.029	82.8	0.047
67.9	0.015	80.9	0.034	82.0	0.049	93.5	0.109	95.6	0.230	87.5	0.358	68.9	0.019	78.5	0.033	78.4	0.050
62.3	0.012	75.2	0.030	72.7	0.045	89.9	0.110	90.4	0.230	81.9	0.349	62.1	0.018	75.0	0.034	73.2	0.053
53.8	0.009	67.9	0.024	65.4	0.037	85.5	0.107	85.1	0.227	74.0	0.341	57.2	0.017	68.2	0.035	67.6	0.054
44.3	0.006	58.2	0.019	58.8	0.029	78.2	0.104	76.0	0.221	67.6	0.331	47.0	0.014	60.5	0.033	63.6	0.052
34.0	0.002	50.6	0.010	51.1	0.021	72.2	0.102	69.1	0.215	58.7	0.300	39.0	0.012	55.5	0.031	57.7	0.050
24.8	0.002	41.5	0.004	42.0	0.013	64.8	0.098	60.8	0.199	54.2	0.279	30.1	0.009	45.2	0.025	51.8	0.047
12.2	0.002	33.1	0.002	33.6	0.007	58.7	0.091	53.1	0.174	46.4	0.224	19.6	0.006	37.2	0.019	44.3	0.039
5.4	0.002	25.1	0.003	25.3	0.003	51.3	0.079	47.8	0.148	41.4	0.179	12.2	0.004	28.7	0.012	38.6	0.031

	16.1	0.002	19.0	0.002	43.9	0.06	40.1	0.103	34.5	0.113	4.5	0.002	21.6	0.007	31.7	0.023
	8.8	0.002	9.5	0.003	37.5	0.042	33.8	0.070	26.5	0.065			15.2	0.004	22.9	0.014
					32.3	0.029	28.5	0.046	17.3	0.027			8.6	0.002	17.0	0.010
					24.4	0.014	22.3	0.028	9.1	0.014					12.3	0.006
					16.5	0.006	17.1	0.016	1.3	0.009					5.9	0.003
					8.4	0.002	10.5	0.008								
							4.7	0.004								

Table S5. The testing data for current-intensity relationship.

Angle: 60°						Angle: 90°						Angle: 120°					
Intensity (W m^{-2})	I_A (μA)	Intensity (W m^{-2})	I_B (μA)	Intensity (W m^{-2})	I_C (μA)	Intensity (W m^{-2})	I_A (μA)	Intensity (W m^{-2})	I_B (μA)	Intensity (W m^{-2})	I_C (μA)	Intensity (W m^{-2})	I_A (μA)	Intensity (W m^{-2})	I_B (μA)	Intensity (W m^{-2})	I_C (μA)
107	0.016	107	0.136	107	0.024	107	0.023	107	0.182	107	0.021	107	0.028	107	0.126	107	0.012
135	0.020	135	0.177	135	0.032	135	0.030	135	0.236	135	0.028	135	0.035	135	0.160	135	0.015
164	0.024	164	0.223	164	0.042	164	0.038	164	0.277	164	0.035	164	0.044	164	0.196	164	0.018
192	0.027	192	0.268	192	0.050	192	0.044	192	0.336	192	0.042	192	0.053	192	0.240	192	0.022
218	0.032	218	0.310	218	0.057	218	0.051	218	0.407	218	0.048	218	0.061	218	0.281	218	0.025
245	0.037	245	0.355	245	0.066	245	0.058	245	0.466	245	0.054	245	0.068	245	0.322	245	0.030
268	0.042	268	0.40	268	0.074	268	0.065	268	0.516	268	0.061	268	0.077	268	0.360	268	0.033
289	0.046	289	0.444	289	0.082	289	0.073	289	0.585	289	0.067	289	0.085	289	0.401	289	0.037
309	0.050	309	0.486	309	0.092	309	0.080	309	0.636	309	0.073	309	0.094	309	0.442	309	0.042
327	0.054	327	0.532	327	0.099	327	0.087	327	0.687	327	0.079	327	0.101	327	0.482	327	0.046

Table S6. The interpolated data for current-angle relationship.

Angle (°)	Intensity: 107 W m^{-2}			Intensity: 201 W m^{-2}			Intensity: 282 W m^{-2}		
	I_A (μA)	I_B (μA)	I_C (μA)	I_A (μA)	I_B (μA)	I_C (μA)	I_A (μA)	I_B (μA)	I_C (μA)
2	0.0012	0	0.0029	0	0.0028	0.0022	0.0018	0.0035	0.0039
8	0.0012	0.0011	0.0027	0	0.0053	0.0028	0.0021	0.0056	0.0048
14	0.0015	0.0032	0.0037	0.0014	0.0092	0.0038	0.0025	0.0148	0.0066
20	0.0019	0.0102	0.0054	0.0018	0.0226	0.0059	0.0037	0.0390	0.0108
26	0.0024	0.0202	0.0075	0.0021	0.0435	0.0102	0.0049	0.0725	0.0175
32	0.0032	0.0319	0.0098	0.0031	0.0684	0.0151	0.0065	0.1117	0.025
38	0.0042	0.0447	0.0120	0.0045	0.0955	0.0200	0.0104	0.1538	0.0325
44	0.0059	0.0576	0.0141	0.0076	0.1230	0.0246	0.0151	0.1961	0.0392
50	0.0078	0.0700	0.0158	0.0112	0.1496	0.0285	0.0204	0.2367	0.0448
56	0.0098	0.0815	0.0171	0.0153	0.1739	0.0314	0.026	0.2737	0.0489
62	0.0119	0.0915	0.0179	0.0196	0.1951	0.0332	0.0319	0.3057	0.0513
68	0.0139	0.0997	0.0182	0.024	0.2125	0.034	0.0377	0.3317	0.0521
74	0.0158	0.1059	0.0181	0.0283	0.2255	0.0336	0.0433	0.3510	0.0513
80	0.0176	0.1099	0.0175	0.0324	0.2338	0.0323	0.0484	0.3631	0.049
86	0.0191	0.1116	0.0165	0.0360	0.2372	0.0301	0.0528	0.3679	0.0454
92	0.0203	0.1111	0.0152	0.0389	0.2359	0.0273	0.0565	0.3653	0.0409
98	0.0211	0.1083	0.0137	0.0411	0.2299	0.0239	0.0592	0.3558	0.0356
104	0.0215	0.1035	0.0120	0.0425	0.2196	0.0203	0.0609	0.3399	0.030
110	0.0216	0.0969	0.0102	0.0428	0.2054	0.0167	0.0613	0.3181	0.0243
116	0.0212	0.0886	0.0084	0.0422	0.1879	0.0132	0.0605	0.2914	0.0189
119	0.0208	0.0840	0.0075	0.0414	0.1781	0.0115	0.0597	0.2765	0.0163
124	0.0200	0.0756	0.0061	0.0397	0.1605	0.009	0.0575	0.2499	0.0125
130	0.0186	0.0648	0.0047	0.0366	0.1379	0.0066	0.0537	0.2157	0.0087
136	0.0168	0.0536	0.0035	0.0327	0.1144	0.0047	0.0487	0.1802	0.0058
142	0.0146	0.0423	0.0026	0.0280	0.0909	0.0035	0.0426	0.1448	0.0038
148	0.0122	0.0315	0.0020	0.0227	0.0682	0.0028	0.0355	0.1108	0.0029
154	0.0097	0.0215	0.0016	0.0172	0.0473	0.0027	0.0276	0.0794	0.0026
160	0.0070	0.0129	0.0016	0.0118	0.0292	0.0027	0.0193	0.0521	0.0028
166	0.0044	0.0061	0.0017	0.0068	0.0147	0.0027	0.0107	0.0301	0.0029
172	0.0019	0.0017	0.0020	0.0029	0.0046	0.0021	0.0051	0.0147	0.0022
178	0	0	0.0022	0	0	0	0	0.0071	0

Table S7. The interpolated data for current-intensity relationship.

Intensity (W m^{-2})	Angle: 60°			Angle: 90°			Angle: 120°		
	I_A (μA)	I_B (μA)	I_C (μA)	I_A (μA)	I_B (μA)	I_C (μA)	I_A (μA)	I_B (μA)	I_C (μA)
100	0.0143	0.1279	0.0226	0.0233	0.1672	0.0221	0.0258	0.1163	0.0102
105	0.0151	0.1363	0.0242	0.0247	0.1780	0.0233	0.0274	0.1238	0.0109
110	0.0160	0.1446	0.0258	0.0260	0.1889	0.0245	0.0289	0.1313	0.0116
115	0.0168	0.1530	0.0274	0.0274	0.1997	0.0257	0.0305	0.1388	0.0124
120	0.0176	0.1613	0.0290	0.0287	0.2106	0.0269	0.0321	0.1463	0.0131
125	0.0184	0.1697	0.0306	0.0300	0.2214	0.0282	0.0336	0.1538	0.0138
130	0.0193	0.1780	0.0321	0.0314	0.2323	0.0294	0.0352	0.1613	0.0145
135	0.0201	0.1864	0.0337	0.0327	0.2431	0.0306	0.0368	0.1688	0.0152
140	0.0209	0.1947	0.0353	0.0341	0.2540	0.0318	0.0383	0.1763	0.0160
145	0.0217	0.2031	0.0369	0.0354	0.2648	0.0331	0.0399	0.1838	0.0167
150	0.0226	0.2114	0.0385	0.0368	0.2757	0.0343	0.0414	0.1913	0.0174
155	0.0234	0.2198	0.0400	0.0381	0.2865	0.0355	0.0430	0.1988	0.0181
160	0.0242	0.2281	0.0416	0.0395	0.2974	0.0367	0.0446	0.2063	0.0188
165	0.0250	0.2365	0.0432	0.0408	0.3082	0.0379	0.0461	0.2138	0.0196
170	0.0259	0.2448	0.0448	0.0422	0.3191	0.0392	0.0477	0.2213	0.0203
175	0.0267	0.2532	0.0464	0.0435	0.3299	0.0404	0.0493	0.2288	0.0210
180	0.0275	0.2615	0.0480	0.0449	0.3408	0.0416	0.0508	0.2363	0.0217
185	0.0284	0.2699	0.0495	0.0462	0.3516	0.0428	0.0524	0.2438	0.0224
190	0.0292	0.2782	0.0511	0.0476	0.3625	0.0440	0.0540	0.2513	0.0232
195	0.0300	0.2866	0.0527	0.0489	0.3733	0.0453	0.0555	0.2588	0.0239
200	0.0308	0.2949	0.0543	0.0502	0.3842	0.0465	0.0571	0.2663	0.0246
205	0.0317	0.3033	0.0559	0.0516	0.3950	0.0477	0.0587	0.2738	0.0253
210	0.0325	0.3116	0.0574	0.0529	0.4059	0.0489	0.0602	0.2813	0.0260
215	0.0333	0.3200	0.0590	0.0543	0.4167	0.0501	0.0618	0.2888	0.0268
220	0.0341	0.3283	0.0606	0.0556	0.4276	0.0514	0.0634	0.2963	0.0275
225	0.0350	0.3367	0.0622	0.0570	0.4384	0.0526	0.0649	0.3038	0.0282
230	0.0358	0.345	0.0638	0.0583	0.4493	0.0538	0.0665	0.3113	0.0289
235	0.0366	0.3534	0.0654	0.0597	0.4601	0.0550	0.0681	0.3188	0.0296
240	0.0374	0.3617	0.0669	0.0610	0.4710	0.0563	0.0696	0.3263	0.0303
245	0.0383	0.3701	0.0685	0.0624	0.4818	0.0575	0.0712	0.3338	0.0311
250	0.0391	0.3784	0.0701	0.0637	0.4927	0.0587	0.0728	0.3413	0.0318
255	0.0399	0.3868	0.0717	0.0651	0.5035	0.0599	0.0743	0.3488	0.0325
260	0.0408	0.3951	0.0733	0.0664	0.5144	0.0611	0.0759	0.3563	0.0332
265	0.0416	0.4035	0.0749	0.0677	0.5252	0.0624	0.0775	0.3638	0.0339

270	0.0424	0.4118	0.0764	0.0691	0.5361	0.0636	0.0790	0.3713	0.0347
275	0.0432	0.4202	0.0780	0.0704	0.5469	0.0648	0.0806	0.3788	0.0354
280	0.0441	0.4285	0.0796	0.0718	0.5578	0.0660	0.0822	0.3863	0.0361
285	0.0449	0.4369	0.0812	0.0731	0.5686	0.0672	0.0837	0.3938	0.0368
290	0.0457	0.4452	0.0828	0.0745	0.5795	0.0685	0.0853	0.4013	0.0375
295	0.0465	0.4536	0.0843	0.0758	0.5903	0.0697	0.0869	0.4088	0.0383
300	0.0474	0.4619	0.0859	0.0772	0.6012	0.0709	0.0884	0.4163	0.0390
305	0.0482	0.4703	0.0875	0.0785	0.612	0.0721	0.0900	0.4238	0.0397
310	0.049	0.4786	0.0891	0.0799	0.6229	0.0734	0.0915	0.4313	0.0404
315	0.0498	0.4870	0.0907	0.0812	0.6337	0.0746	0.0931	0.4388	0.0411
320	0.0507	0.4953	0.0923	0.0826	0.6446	0.0758	0.0947	0.4463	0.0419
325	0.0515	0.5037	0.0938	0.0839	0.6554	0.0770	0.0962	0.4538	0.0426
330	0.0523	0.5120	0.0954	0.0852	0.6663	0.0782	0.0978	0.4613	0.0433

Table S8. The fitted angle and the error between the fitted angle and interpolated angle.

Interpolated angle (°)	Intensity: 107 W m^{-2}		Intensity: 201 W m^{-2}		Intensity: 282 W m^{-2}	
	Fitted angle (°)	Error (°)	Fitted angle (°)	Error (°)	Fitted angle (°)	Error (°)
2	-8.58	10.58	17.42	15.42	26.58	24.58
8	29.56	21.56	7.78	0.22	0.38	7.62
14	5.76	8.24	20.61	6.61	12.26	1.74
20	25.35	5.35	29.23	9.23	41.29	11.29
26	23.66	2.34	10.98	15.02	18.97	7.03
32	28.32	3.68	31.77	0.23	33.49	1.49
38	44.56	6.56	31.03	6.97	40.36	2.36
44	43.26	0.74	47.37	3.37	45.20	1.20
50	47.81	2.19	53.36	3.36	47.85	2.15
56	54.83	1.17	53.23	2.77	52.54	3.46
62	63.86	1.86	62.76°	0.76°	64.73	2.73
68	67.51	0.49	70.06	2.06	69.79	1.79
74	72.48	1.52	80.14	6.14	77.12	3.12
80	79.09	0.91	82.07	2.07	79.95	0.05
86	84.36	1.64	83.78	2.22	82.00	4.00
92	91.78	0.22	88.11	3.89	93.61	1.61
98	100.51	2.51	98.91	0.91	98.40	0.40
104	106.36	2.36	106.04	2.04	106.81	2.81

110	110.61	0.61	113.63	3.63	107.53	2.47
116	114.36	1.64	112.55	3.45	108.45	7.55
119	115.74	3.26	119.53	0.53	121.64	2.64
124	122.73	1.27	127.91	3.91	126.87	2.87
130	132.81	2.81	121.19	8.81	126.15	3.85
136	139.00	3.00	141.75	5.75	136.87	0.87
142	139.35	2.65	147.97	5.97	142.41	0.41
148	140.15	7.85	144.19	3.81	148.36	0.36
154	152.37	1.63	150.35	3.65	147.87	6.13
160	161.59	1.59	162.22	2.22	164.44	4.44
166	171.73	5.73	169.81	3.81	166.01	0.01
172	157.56	14.44	176.26	4.26	155.56	16.44
178	176.62	1.38	184.21	6.21	173.12	4.88

Table S9. The fitted intensity and the error between the fitted intensity and interpolated intensity.

Interpolated intensity (W m^{-2})	Angle: 60°		Angle: 90°		Angle: 120°	
	Fitted intensity (W m^{-2})	Error (%)	Fitted intensity (W m^{-2})	Error (%)	Fitted intensity (W m^{-2})	Error (%)
100	108.08	8.08	95.73	4.27	103.53	3.53
105	106.34	1.28	99.61	5.13	111.55	6.24
110	113.39	3.08	107.53	2.25	112.28	2.08
115	113.63	1.20	111.18	3.32	107.57	6.46
120	114.03	4.98	118.49	1.26	116.93	2.56
125	115.83	7.33	128.32	2.66	119.75	4.20
130	130.97	0.75	131.68	1.29	129.12	0.68
135	133.22	1.32	138.01	2.23	138.30	2.45
140	135.30	3.36	141.29	0.92	131.31	6.21
145	138.32	4.61	149.87	3.36	140.78	2.91
150	146.91	2.06	153.01	2.01	144.79	3.47
155	155.51	0.33	158.72	2.40	153.97	0.67
160	158.34	1.04	161.85	1.16	162.91	1.82
165	161.84	1.91	167.39	1.45	158.49	3.95
170	170.13	0.08	172.69	1.58	167.43	1.51
175	173.75	0.71	178.03	1.73	176.13	0.64
180	177.05	1.64	181.15	0.64	180.36	0.20
185	189.77	2.58	186.40	0.76	188.74	2.02
190	192.99	1.57	189.57	0.23	189.77	0.12
195	196.69	0.87	196.65	0.85	194.06	0.48

200	200.06	0.03	201.83	0.92	202.14	1.07
205	208.02	1.47	204.97	0.02	210.01	2.44
210	215.06	2.41	210.14	0.07	214.11	1.96
215	218.72	1.73	213.38	0.75	215.52	0.24
220	222.07	0.94	220.24	0.11	223.09	1.41
225	229.55	2.02	223.56	0.64	227.16	0.96
230	232.86	1.24	228.74	0.55	234.46	1.94
235	236.51	0.65	232.20	1.19	241.59	2.80
240	243.01	1.26	238.98	0.43	245.46	2.28
245	250.05	2.06	242.56	1.00	247.03	0.83
250	253.28	1.31	247.84	0.87	253.89	1.56
255	256.80	0.71	251.58	1.34	257.69	1.06
260	263.27	1.26	256.95	1.17	264.32	1.66
265	266.73	0.65	263.70	0.49	270.81	2.19
270	272.67	0.99	267.73	0.84	269.67	0.12
275	276.06	0.39	273.17	0.67	276.05	0.38
280	282.17	0.78	277.39	0.93	282.30	0.82
285	285.49	0.17	282.96	0.72	285.86	0.30
290	288.56	0.50	288.69	0.45	291.91	0.66
295	294.36	0.22	294.40	0.21	293.54	0.50
300	300.14	0.05	299.00	0.33	297.01	1.00
305	303.33	0.55	304.88	0.04	302.85	0.71
310	306.27	1.20	310.95	0.31	306.23	1.22
315	309.44	1.77	316.98	0.63	311.90	0.99
320	303.74	5.08	323.09	0.97	321.95	0.61
325	325.71	0.22	322.16	0.87	324.74	0.08
330	330.97	0.29	318.28	3.55	328.22	0.54

Supplementary Figures

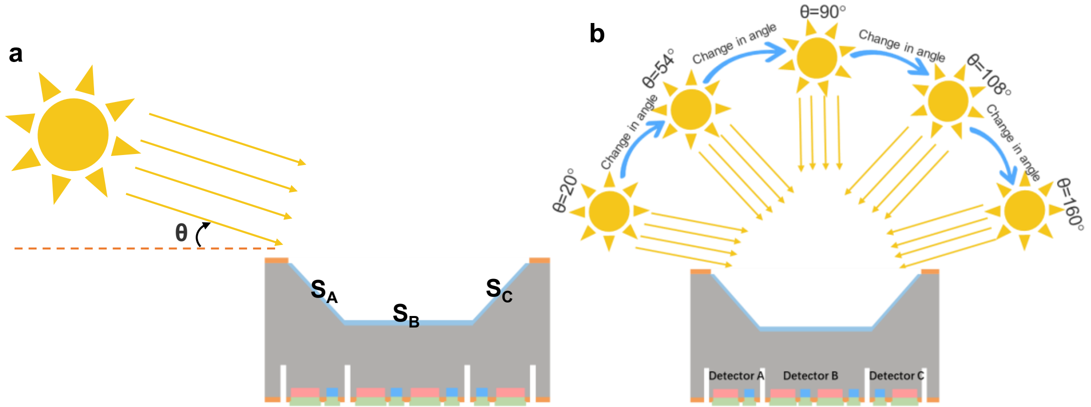

Fig. S3. The definition of the angle of incident light. (a) The light is incident at an angle of θ. (b) The incident light from different angles.

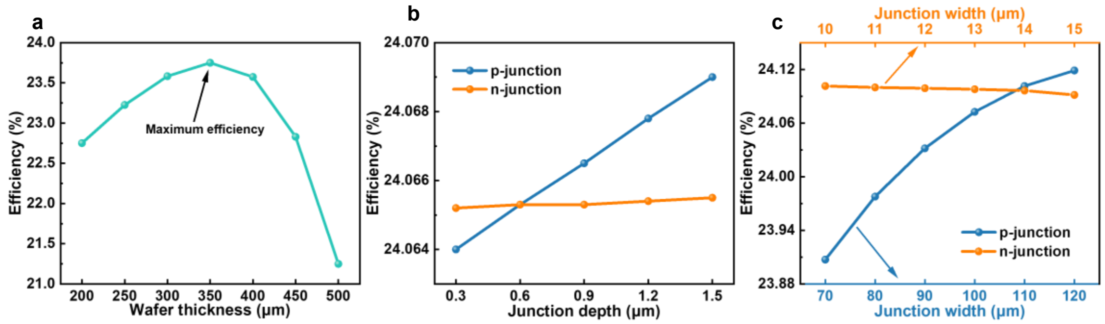

Fig. S4. The simulation results based on Quokka2.2.5. The impact of (a) the wafer thickness, as well as (b) the depth and (c) the width of p-n junction on the efficiency of the photodiode.

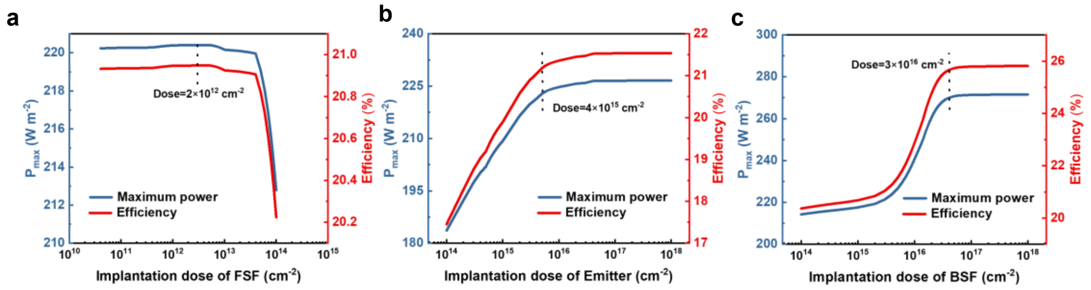

Fig. S5. The implantation dose simulation results for a pn junction in the device based on Silvaco TCAD simulation. The impact of the (a) FSF, (b) Emitter electrode, and (c) BSF implantation dose for Detector A.

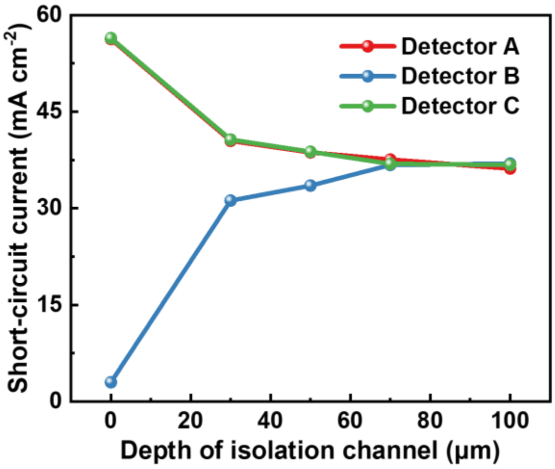

Fig. S6. The simulation of the impact of the depth of the isolation channel for the MISS device.

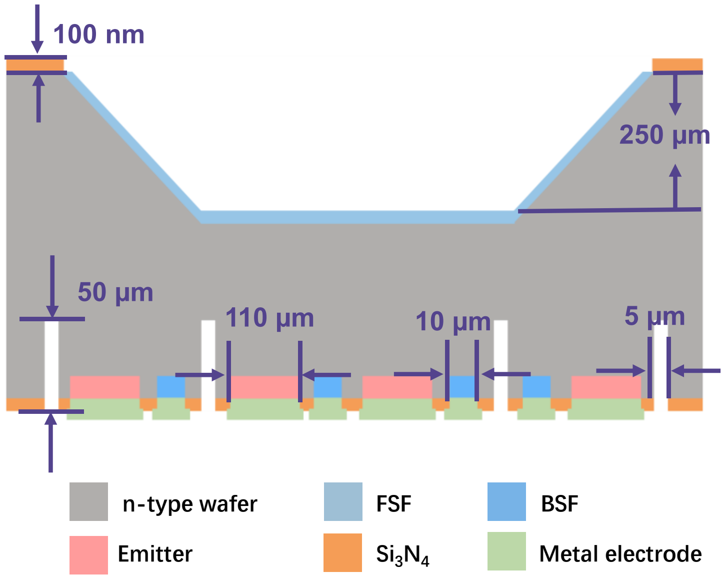

Fig. S7. Basic structural parameters of the MISS device.

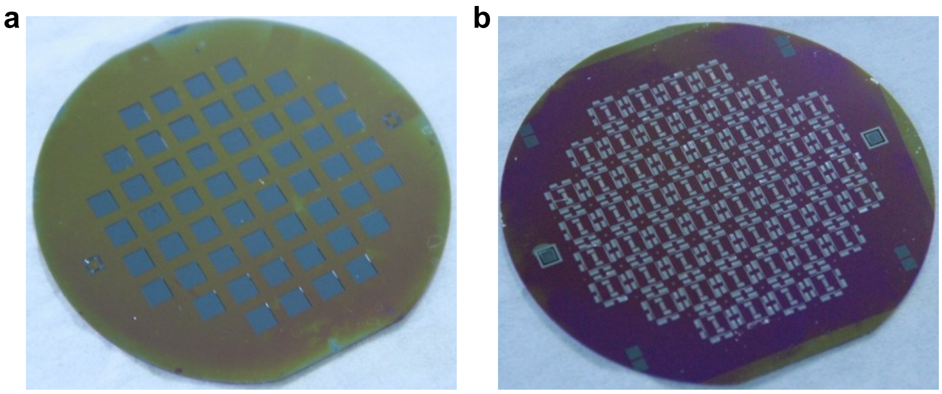

Fig. S8. The optical photos of the fabricated wafer after completing the whole process. (a) The front surface and (b) back surface.

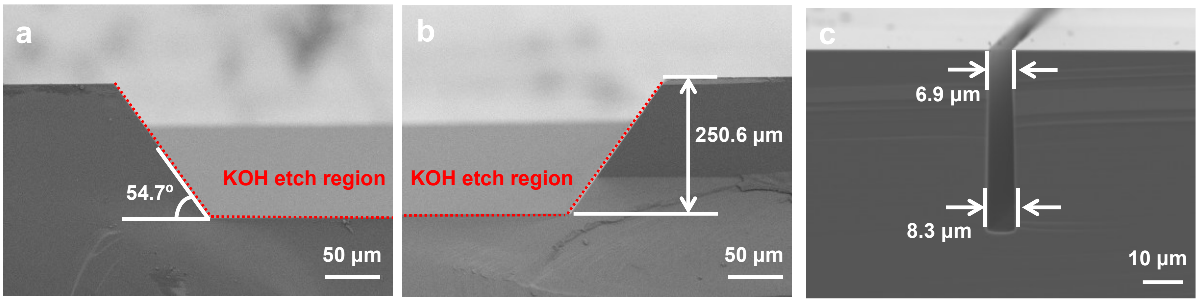

Fig. S9. The detail SEM images of the MISS device. (a) The left inclined surface. (b) The right inclined surface. (c) The isolation channel.

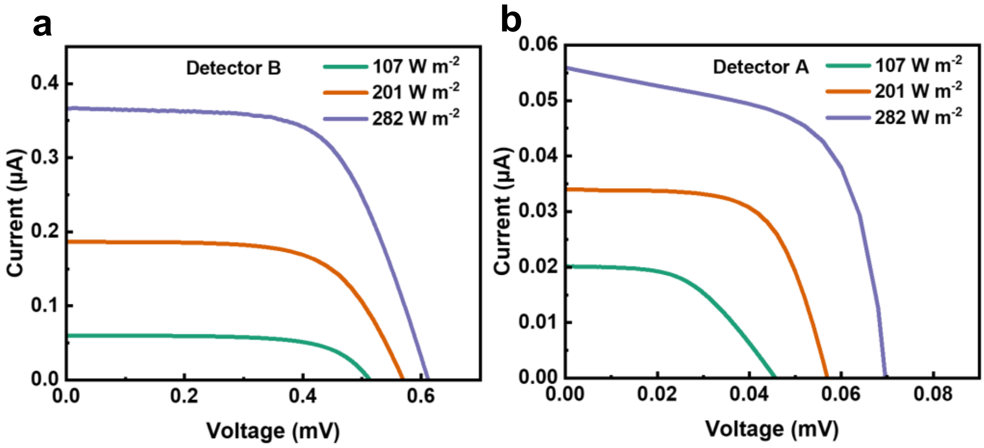

Fig. S10. I-V curves of (a) the detector B and (b) the detector A under various intensities.

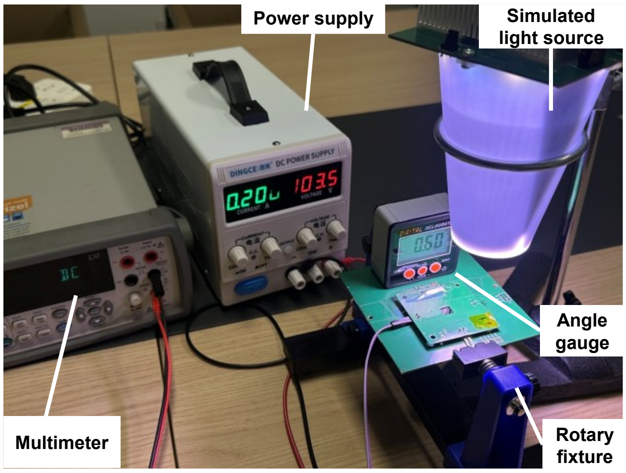

Fig. S11. Photograph of the experimental test setup.

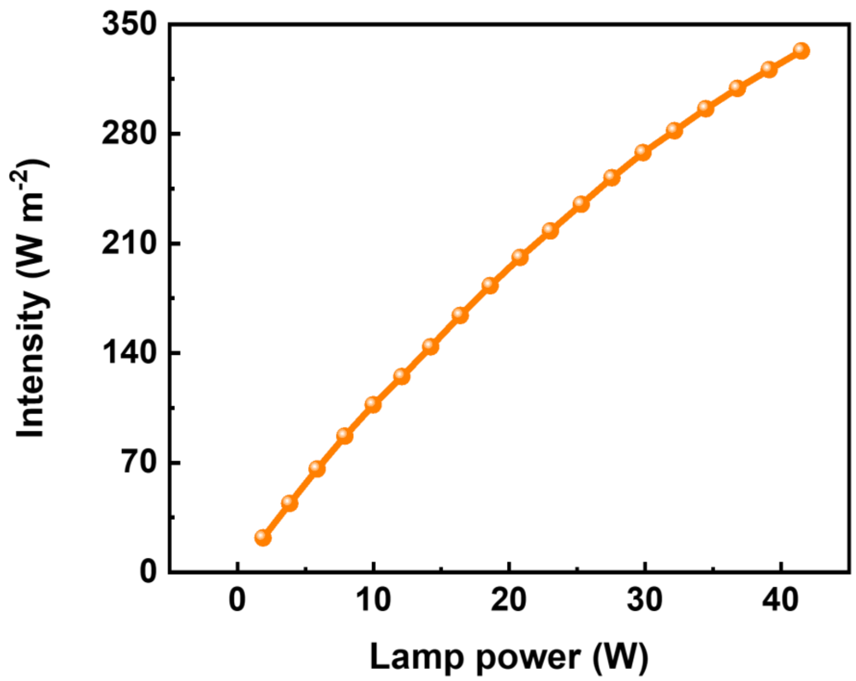

Fig. S12. The relationship between lamp power and intensity.

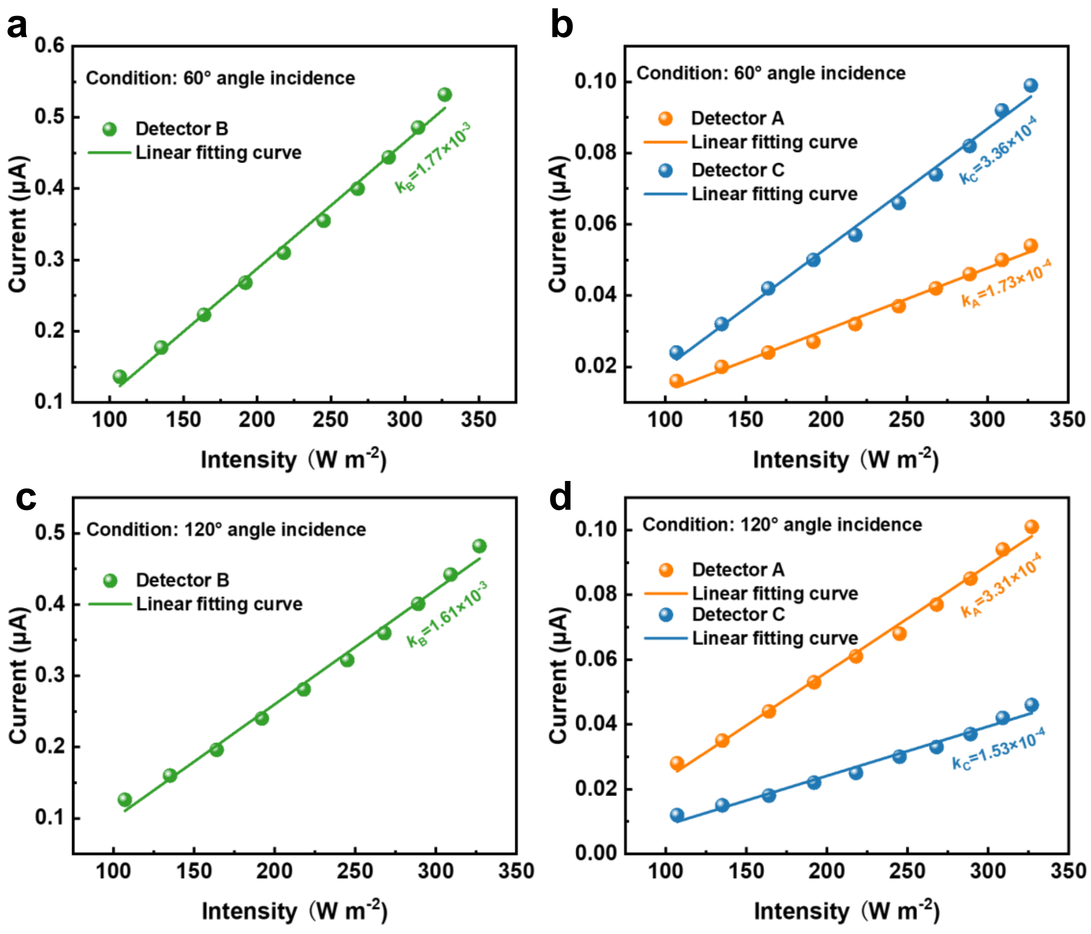

Fig. S13. The relationship between intensity and output current. (a, b) The light is 60° angle

incidence. (c, d) The light is 120° angle incidence.

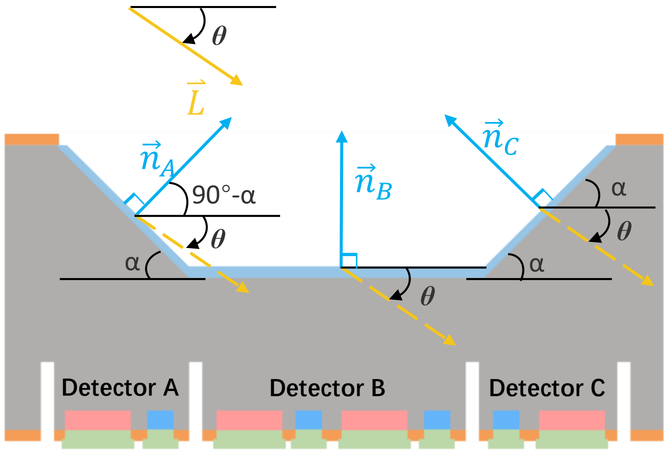

Fig. S14. The physical model of light sensing for MISS device.

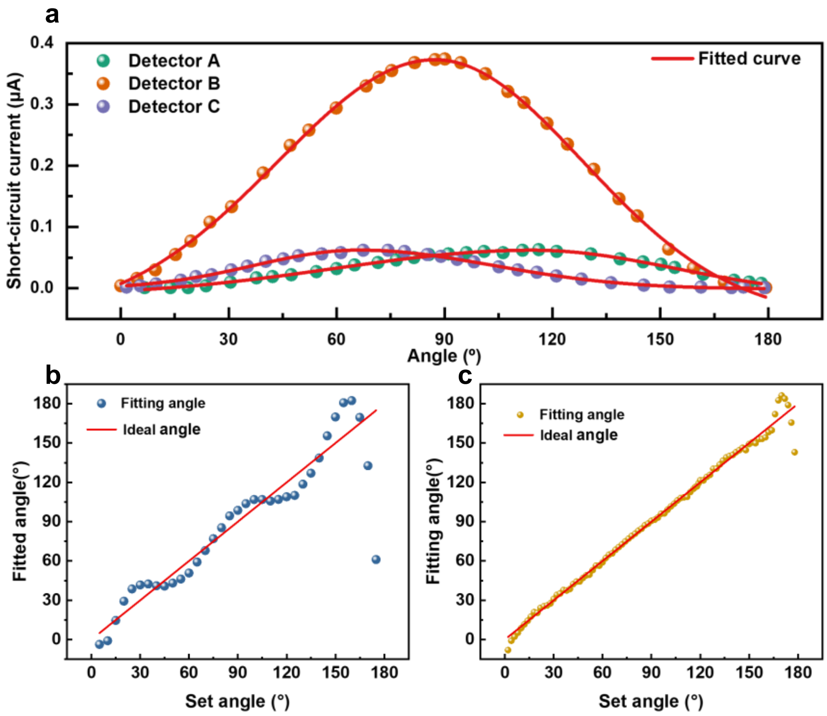

Fig. S15. Fitting analysis for solar angle. (a) The experimental data. (b) The fitting angle based on a basic model without accounting for losses and carrier drift. (c) The fitting angle based on the revised model.

Supplementary Material References

1 Gu, S. *et al.* Tin and Mixed Lead-Tin Halide Perovskite Solar Cells: Progress and their Application in Tandem Solar Cells. *Adv. Mater.* **32**, 16 (2020). Doi: 10.1002/adma.201907392.

2 Oni, A. M., Mohsin, A., Rahman, M. M. & Bhuian, M. B. H. A Comprehensive Evaluation of Solar Cell Technologies, Associated Loss Mechanisms, and Efficiency Enhancement Strategies for Photovoltaic Cells. *Energy Rep.* **11**, 3345-3366 (2024). Doi: 10.1016/j.egyr.2024.03.007.

3 Burlingame, Q., Ball, M. & Loo, Y. L. It's Time to Focus on Organic Solar Cell Stability. *Nat. Energy* **5**, 947-949 (2020). Doi: 10.1038/s41560-020-00732-2.

4 Bartesaghi, D. *et al.* Competition between Recombination and Extraction of Free Charges Determines the Fill Factor of Organic Solar Cells. *Nat. Commun.* **6**, 10 (2015). Doi: 10.1038/ncomms8083.

5 Chiang, C. H. & Wu, C. G. Bulk Heterojunction Perovskite-PCBM Solar Cells with High Fill Factor. *Nat. Photonics* **10**, 196 (2016). Doi: 10.1038/nphoton.2016.3.

6 Anctil, A. & Fthenakis, V. Critical Metals in Strategic Photovoltaic Technologies: Abundance Versus Recyclability. *Prog. Photovolt: Res. Appl.* **21**, 1253-1259 (2013). Doi: 10.1002/pip.2308.

7 Fell, A., Fong, K. C., McIntosh, K. R., Franklin, E. & Blakers, A. W. 3-D Simulation of Interdigitated-Back-Contact Silicon Solar Cells With Quokka Including Perimeter Losses. *IEEE J. Photovolt.* **4**, 1040-1045 (2014). Doi: 10.1109/jphotov.2014.2320302.

8 Dosopoulos, S. & Lee, J. F. Interior Penalty Discontinuous Galerkin Finite Element Method for the Time-Dependent First Order Maxwell's Equations. *IEEE Trans. Antennas Propag.* **58**, 4085-4090 (2010). Doi: 10.1109/tap.2010.2078445.

9 Volovichev, I. N., Velázquez-Pérez, J. E. & Gurevich, Y. G. New Boundary Conditions for the Study of Charge Transport in Solid-State Devices. In *26th International Conference on Microelectronics.* 151 (Ieee, 2008).

10 Anta, J. A. *et al.* A Continuity Equation for the Simulation of the Current-Voltage Curve and the Time-Dependent Properties of Dye-Sensitized Solar Cells. *Phys. Chem. Chem. Phys.* **14**, 10285-10299 (2012). Doi: 10.1039/c2cp40719a.

11 Keymasi, F., Keshavarz, A. & Hatami, M. The Effect of Porosity on the Current Electron Density at Dye Solar Cell. *Optik* **126**, 2539-2542 (2015). Doi: 10.1016/j.ijleo.2015.06.042.

12 Dumitru, C., Muscurel, V., Nordseth, O., Fara, L. & Sterian, P. Optimization of Electro-Optical Performance and Material Parameters for a Tandem Metal Oxide Solar Cell. In *18th International Conference on Computational Science and Its Applications (ICCSA).* 573-582 (Springer International Publishing Ag, 2018).

13 Zhang, Y. A., Stokes, N., Jia, B., Fan, S. H. & Gu, M. Towards Ultra-Thin Plasmonic Silicon Wafer Solar Cells with Minimized Efficiency Loss. *Sci. Rep.* **4**, 6 (2014). Doi: 10.1038/srep04939.

14 Yoshiba, S., Hirai, M., Abe, Y., Konagai, M. & Ichikawa, Y. Single Crystalline Silicon Solar cells with Rib Structure. *AIP Adv.* **7**, 6 (2017). Doi: 10.1063/1.4976721.

15 Asmontas, S., Masalskyi, O., Zharchenko, I., Suziedelis, A. & Gradauskas, J. Some Aspects of Hot Carrier Photocurrent across GaAs p-n Junction. *Inorganics* **12**, 9 (2024). Doi: 10.3390/inorganics12060174.

16 Wei, Z. Q., Al-Nuaimi, N. & Gemming, S. Optimization of InGaN-Based Solar Cells by Numerical Simulation: Enhanced Efficiency and Performance Analysis. *Next Mater.* **6**, 11 (2025). Doi: 10.1016/j.nxmate.2024.100325.

17 Hak Kee, J., Taniguchi, K. & Hamaguchi, C. Theoretical Study of Minority Carrier Lifetimes Clue to Auger Recombination in n-Type silicon. *Jpn. J. Appl. Phys. 1, Regul. Pap. Short Notes (Japan)* **34**, 3054-3058 (1995). Doi: 10.1143/jjap.34.3054.

18 Tanaka, K., Nagaya, K. & Kato, M. 4H-SiC Auger Recombination Coefficient under the High Injection Condition. *Jpn. J. Appl. Phys.* **62**, 4 (2023). Doi: 10.35848/1347-4065/acaca8.

19 Alamgeer *et al.* Improved Passivation and Antireflection Techniques for Higher-Efficiency Interdigitated Back Contact (IBC) Solar Cells. *J. Comput. Electron.* **24**, 12 (2025). Doi: 10.1007/s10825-025-02289-3.

20 Peng, J. B. *et al.* Interdigitated Back Contact (IBC) GaSb Thermophotovoltaic Cell on Silicon Substrate. *Infrared Phys. Technol.* **147**, 9 (2025). Doi: 10.1016/j.infrared.2025.105789.

21 Acharyya, S., Ghosh, D. K., Banerjee, D. & Maity, S. Analyzing the Operational Versatility of Advanced IBC Solar Cells at Different Temperatures and also with Variation in Minority Carrier Lifetimes. *J. Comput. Electron.* **23**, 1170-1194 (2024). Doi: 10.1007/s10825-024-02232-y.

22 Kuruganti, V. V., Isabella, O. & Mihailetchi, V. D. Structuring Interdigitated Back Contact Solar Cells Using the Enhanced Oxidation Characteristics Under Laser-Doped Back Surface Field Regions. *Phys. Status Solidi A-Appl. Mat.* **221**, 7 (2024). Doi: 10.1002/pssa.202300820.

23 Yang, X. Y., Tu, Y. G., Ye, F. J. & Bao, Z. Back-Contact Configuration Energizes Perovskite Photovoltaic Modules. *Nano Res. Energy* **3**, 5 (2024). Doi: 10.26599/nre.2024.9120111.

24 Mertens, V. *et al.* Plasma-Enhanced Chemical-Vapor-Deposited $SiO_x(N_y)$/n-type Polysilicon-on-Oxide-Passivating Contacts in Industrial Back-Contact Si Solar Cells. *Sol. RRL* **8**, 9 (2024). Doi: 10.1002/solr.202300919.

25 Gao, J. Q. *et al.* Over 700 mV IBC Solar Cell by Optimizing Front Surface Field Passivation. *IEEE J. Photovolt.* **13**, 56-60 (2023). Doi: 10.1109/jphotov.2022.3229528.

26 Jiang, K. *et al.* Triple-Layered nc-Si:H Films Improve Electrical Properties and Expand Process Window of IBC-SHJ Solar Cells Simulated by Silvaco TCAD. *Sci. China-Mater.* **66**, 4891-4896 (2023). Doi: 10.1007/s40843-023-2610-y.

27 Fazal, M. A. & Rubaiee, S. Progress of PV Cell Technology: Feasibility of Building Materials, Cost, Performance, and Stability. *Sol. Energy* **258**, 203-219 (2023). Doi: 10.1016/j.solener.2023.04.066.

28 Mita, Y. *et al.* Progress and Opportunities in High-Voltage Microactuator Powering Technology towards One-Chip MEMS. *Jpn. J. Appl. Phys.* **57**, 14 (2018). Doi: 10.7567/jjap.57.04fa05.

29 Shahnooshi, F. & Orouji, A. A. Efficiency Improvement of Graphene/AlGaAs/GaAs Schottky Junction Solar Cells by Minimizing Optical Losses through front and Rear Surface Texturing. *Sci. Rep.* **15**, 13 (2025). Doi: 10.1038/s41598-025-07080-9.

30 Xiong, Q. *et al.* Managed Spatial Strain Uniformity for Efficient Perovskite Photovoltaics Enables Minimized Energy Deficit. *Joule* **8**, 19 (2024). Doi: 10.1016/j.joule.2024.01.016.

31 Hou, F. H. *et al.* Dual Interface Strategies Enable Efficient Wide Bandgap Perovskite Solar Cells. *Appl. Phys. Lett.* **124**, 7 (2024). Doi: 10.1063/5.0189968.

32 Rhaim, Z., Echouchene, F., Habli, S. & Gazzah, M. H. Enhancing the Efficiency of Silicon Solar Cells: An Optimization Approach Using the Taguchi Method and Artificial Neural Networks. *2024 IEEE International Conference on Artificial Intelligence & Green Energy (ICAIGE)*, 1-5 (2024). Doi: 10.1109/icaige62696.2024.10776634.

33 Liu, J. H., Zhang, F. & Huang, H. J. Research Progress on Illumination System Technology of Step-and-Scan Projection Lithography Tools. *Laser Optoelectron. Prog.* **59**, 9 (2022). Doi: 10.3788/lop202259.0922011.

34 Cheng, X. Y. *et al.* Review of Directed Self-Assembly Material, Processing, and Application in Advanced Lithography and Patterning. *Micromachines* **16**, 19 (2025). Doi: 10.3390/mi16060667.

35 Zheng, L., Reinhardt, C. & Roth, B. Microscope Projection Photolithography-Enabled Structuring with Subwavelength Resolution. *2023 Conference on Lasers and Electro-Optics Europe & European Quantum Electronics Conference (CLEO/Europe-EQEC)*, 1-1 (2023). Doi: 10.1109/CLEO/Europe-EQEC57999.2023.10231498.
